# The Proteome of *Dictyostelium discoideum* Across Its Entire Life Cycle Reveals Sharp Transitions Between Developmental Stages

**DOI:** 10.3390/proteomes14010003

**Published:** 2026-01-08

**Authors:** Sarena Banu, P. V. Anusha, Pedro Beltran-Alvarez, Mohammed M. Idris, Katharina C. Wollenberg Valero, Francisco Rivero

**Affiliations:** 1 Centre for Biomedicine, Hull York Medical School, Faculty of Health Sciences, University of Hull, Hull HU6 7RX, UK; s.nagoor-pitchai-2021@hull.ac.uk (S.B.); pedro.beltran-alvarez@hyms.ac.uk (P.B.-A.); 2 Energy and Environment Institute, University of Hull, Hull HU6 7RX, UK; 3 CSIR-Centre for Cellular and Molecular Biology, Uppal Road, Hyderabad 500007, Telangana, India; anusha.ccmb@csir.res.in (P.V.A.); idris.ccmb@csir.res.in (M.M.I.); 4 School of Biology and Environmental Science, University College Dublin, D04 V1W8 Dublin, Ireland; katharina.wollenbergvalero@ucd.ie; 5 Conway Institute, University College Dublin, D04 V1W8 Dublin, Ireland

**Keywords:** development, *Dictyostelium discoideum*, label-free quantification, mass spectrometry, proteomics, hierarchical clustering, functional enrichment

## Abstract

Background: *Dictyostelium discoideum* is widely used in developmental and evolutionary biology due to its ability to transition from a single cell to a multicellular organism in response to starvation. While transcriptome information across its life cycle is widely available, only early-stage data exist at the proteome level. This study characterizes and compares the proteomes of *D. discoideum* cells at the vegetative, aggregation, mound, culmination and fruiting body stages. Methods: Samples were collected from cells developing synchronously on nitrocellulose filters. Proteins were extracted and digested with trypsin, and peptides were analyzed by liquid chromatography–tandem mass spectrometry. Data were processed using Proteome Discoverer™ for protein identification and label-free quantification. Results: A total of 4502 proteins were identified, of which 1848 (41%) were present across all stages. Pairwise comparisons between adjacent stages revealed clear transitions, the largest ones occurring between the culmination and fruiting body and between the fruiting body and vegetative stage, involving 29% and 52% of proteins, respectively. Hierarchical clustering assigned proteins to one of nine clusters, each displaying a distinct pattern of abundances across the life cycle. Conclusions: This study presents the first complete developmental proteomic time series for *D. discoideum*, revealing changes that contribute to multicellularity, cellular differentiation and morphogenesis.

## 1. Introduction

*Dictyostelium discoideum*, an important eukaryotic organism widely used as a model to study the unicellular-to-multicellular transition, has made fundamental contributions to our knowledge of aggregation, differentiation and morphogenesis processes [1]. *D. discoideum* lives as single cells that feed on bacteria by phagocytosis and proliferate through mitotic division. When nutrients are depleted, cells undergo a change in their transcriptional program, reduce cell proliferation and begin to secrete the chemoattractant cyclic AMP (cAMP) in an oscillatory pattern to communicate with surrounding cells. Cells aggregate into groups of 10^5^ cells called mounds and differentiate into two main populations, prespore (approximately 80%) and prestalk (approximately 20%) cells. Most prestalk cells migrate to the tip of the mound, which extends upward to form an early culminant. The culminant develops into a mature fruiting body with a spore head (sorus) and a stalk. Stalk cells become vacuolated and die during differentiation, providing structural support to elevate the fruiting body and facilitate spore dispersal. Spores withstand harsh environmental conditions and remain dormant. When spores meet suitable conditions, they germinate and restart the life cycle [1,2,3].

Numerous transcriptomics studies have been carried out with various degrees of temporal resolution to gain a comprehensive view of gene expression changes across different stages of the life cycle of *D. discoideum*. Those studies revealed the transcriptional fingerprint of each of the main morphological transitions, and identified and annotated milestone genes [4,5,6,7,8]. Collectively, transcriptomics studies have shown that cellular priorities shift during the life cycle. For example, the unicellular stage is associated with protein biosynthesis and metabolic activity, and the multicellular stage involves a process related to adhesion, differentiation and development. Single-cell transcriptomics has allowed a detailed analysis of the first fate choice cells make in the aggregate, namely, the transition into prespore or prestalk cells [9]. Another single-cell transcriptomics study has revealed the unique signatures of mature cup, stalk and spore cells [10]. The unicellular-to-multicellular transition is commonly identified as the one with the greatest transcriptome changes, and developmental time series have also revealed that transcriptional and morphological changes do not always match. For example, the striking changes that occur during culmination are accompanied by few transcriptional changes [5,6].

While several transcriptomics studies have addressed the full life cycle of *D. discoideum*, proteomes across all developmental stages are still missing. Studies have focused on specific cellular components or on specific stages of the life cycle. Sobczyk et al. (2014) performed a detailed analysis of the cytoskeletal fraction of starved cells in response to cAMP in a second-to-minute timescale to investigate the cytoskeleton dynamics during the chemotactic response [11]. Studies using similar conditions have looked at proteins of the peripheral membrane fraction [12] or at the phosphoproteome [13]. A mitochondrial proteomics study showed that the bioenergetic activity of this organelle decreases during the early stages of development, but the full life cycle was not investigated in this study [14]. Proteomics studies of the prespore secretory vesicles [15], the slime sheath [16] and the secretome [17,18,19] have also been undertaken. Wang et al. (2021) addressed histone modifications across the entire developmental cycle and reported significant changes upon the transition to multicellularity [7].

Three full proteome studies in *D. discoideum* have been published; however, they only address early developmental stages. Gonzalez-Velasco et al. (2019) compared the proteomes of vegetative cells and cells starved and pulsed with cAMP for 5.5 h and identified a signature of 136 proteins, 110 of them novel, related to cAMP signaling [20]. Kelly et al. (2021) compared the proteomes of vegetative cells and cells starved for 8 h as part of a wider study but do not provide an analysis of the data [21]. More recently Edelbroek et al. (2024) analyzed RNA-seq data and protein abundances in a developmental time series up to the 10 h mound stage, and reported a high correlation during the unicellular growth phase that dropped as cells progressed into multicellular development, with protein profiles being delayed 2 to 4 h relative to the corresponding transcript profiles [8]. A delay between protein and mRNA profiles has been reported in *D. discoideum* [5,6] and poor correlations between mRNA and protein expressions have been described in several eukaryote development studies [22,23,24,25]. This is important, because it implies that proteomics provides a more accurate understanding of cellular activities and morphological and functional transitions during development than transcriptomics alone. No cellular proteomics study has investigated the complete life cycle of *D. discoideum*. To bridge this knowledge gap, we undertook a full non-labeling proteomics analysis of cells developed synchronously on nitrocellulose filters, covering the vegetative, aggregation, mound, culmination and fruiting body stages. Differential abundance and pairwise comparison analyses identified stage-specific proteins, developmental transitions and a set of proteins stably maintained throughout the *D. discoideum* life cycle.

## 2. Materials and Methods

### 2.1. Cultivation and Development of D. discoideum

Wild type *D. discoideum* AX2 strain, an axenically growing derivative of wild strain NC4, was used for this study. Cells were grown in AX medium (Formedium, Swaffham, UK) in shaking suspension at 22 °C until mid-log phase (1–3 × 10^6^ cells/mL) [26,27]. Cells were harvested, washed twice in Soerensen buffer (2 mM Na_2_HPO_4_ and 15 mM KH_2_PO_4_, pH 6.0) and deposited on 47 mm MF Millipore mixed cellulose ester filters (Merck, Gillingham, UK) (1 × 10^8^ cells per filter) for synchronous development [26,27,28]. Developmental stages were monitored and imaged with a ZEISS SteREO Discovery.V8 stereomicroscope (ZEISS, Oberkochen, Germany).

### 2.2. Mass Spectrometry

Vegetative cells (0 h) were collected immediately after depositing on filters, and aggregation (8 h), mound (12 h), culmination (20 h) and fruiting body (28 h) stages were collected based on developmental morphology. Biological replicates were collected from two experiments performed on different days. Cells were scraped off the filters, washed twice with Soerensen buffer, immediately lysed using homogenization buffer (7 M urea, 2 M thiourea, 18 mM Tris HCl, 4% CHAPS, 14 mM Trizma base, 0.2% Triton X-100, 50 mM dithiothreitol and Complete™ ethylenediaminetetraacetic acid (EDTA)-free protease inhibitor cocktail (Roche Diagnostics GmbH, Mannheim, Germany), pH 7.4), snap-frozen and stored at −80 °C. Samples were later thawed and centrifuged at 20,000× *g* for 30 min at 4 °C to separate cell debris [29,30]. Supernatants were collected and proteins quantified using the Amido Black assay [31]. A total of 100 µg of protein was mixed with 5× loading dye (10% SDS, 500 mM DTT, 50% glycerol, 250 mM Tris-HCl, 0.5% bromophenol blue, pH 6.8), heated at 95 °C for 5 min and resolved per lane on a 12% sodium dodecyl sulfate-polyacrylamide gel and stained with Coomassie blue (Figure S1). To avoid cross-lane mixing, empty lanes were left between samples, and each developmental stage was run on a separate gel (Figure S1). Each lane was sliced with a sterile razor blade into three fractions (high, medium and low molecular weight) to improve peptide recovery and coverage across different protein sizes. Fractions were minced into small pieces and transferred to low-binding microcentrifuge tubes. Gel pieces were destained with destaining solution (50% acetonitrile and 40 mM ammonium bicarbonate), and then washed with nuclease-free water followed by 100% acetonitrile and digested with 400 ng of Trypsin Gold (10 ng/µL) (Promega, Southampton, UK) overnight at 37 °C following the manufacturer’s instructions [32]. The three fractions of each sample were processed separately and their MS results subsequently combined.

Digested peptides were eluted with elution buffer (50% acetonitrile and 0.1% trifluoroacetic acid) and completely dried in a SpeedVac at 30 °C. Peptides were purified and desalted using Pierce™ C18 spin columns according to the manufacturer protocol (ThermoFisher Scientific, Waltham, MA, USA). Desalted peptides were further dried and dissolved in 5% acetonitrile and 0.2% formic acid and processed by liquid chromatography–tandem mass spectrometry (LC-MS/MS) on a Q Exactive HF Orbitrap mass spectrometer coupled to an EASY-nLC 1200 system (ThermoFisher Scientific, Waltham, MA, USA), using a 90 min gradient [33]. Peptide separation was performed using an EASY-Spray™ PepMap RSLC C18 column (150 mm × 75 µm inner diameter, 3 µm particle size, 100 Å pore size) (ThermoFisher Scientific, Waltham, MA, USA).

Data were acquired in data-dependent acquisition mode using a Top10 method. Full MS scans were acquired at a resolution of 70,000 (at *m*/*z* 200) over a scan range of *m*/*z* 400–1750, with an automated gain control target of 3 × 10^6^ and a maximum injection time of 100 ms. The ten most intense precursor ions were selected for higher-energy collisional dissociation fragmentation using a normalized collision energy of 32. MS/MS spectra were acquired at a resolution of 17,500 with an AGC target of 1 × 10^5^ and a maximum injection time of 100 ms, using an isolation window of 2.0 *m*/*z*. Dynamic exclusion was set to 20 s.

### 2.3. Protein Identification

Raw MS data were analyzed using Sequest HT in Proteome Discoverer™ 2.2.3 (ThermoFisher Scientific, Waltham, MA, USA) against the *D. discoideum* database downloaded from NCBI in April 2024. Label-free quantification (LFQ) was performed using the Minora Feature Detector node integrated in Proteome Discoverer™ 2.2.3 (ThermoFisher Scientific, Waltham, MA, USA), which detects chromatographic features across all runs, aligns them based on retention time and mass-to-charge ratio (*m*/*z*) and quantifies precursor ion intensities. Protein abundance was calculated by adding up the normalized intensities of all unique peptides assigned to each protein group. The Precursor Ion Quantifier node was used for normalization across samples to correct for injection and instrument variability. The false discovery rate (FDR) and XCorr (score vs. charge) thresholds were set at 1%. A minimum of one peptide was sufficient for canonical protein identification. All protein IDs were initially mapped to gene IDs using DictyBase. For mapped proteins lacking a gene ID, BLAST+ (v2.16.0) searches were performed against the NCBI non-redundant database restricted to *D. discoideum*. Duplicate protein entries were consolidated under single gene IDs and their peptide abundances added up. Identical proteins encoded by retrotransposable elements DIRS1 and TRE5 that mapped to different gene IDs were manually consolidated under single representative gene IDs and their peptide abundances added up. The consolidated raw data can be found in Supplementary spreadsheet S1.

### 2.4. Data Analysis

All downstream statistical analysis and visualizations were performed in R version 4.3.3 [34]. Missing abundance values of biological replicates affected between 2.31% and 14.32% of proteins depending on the stage (Table S1) and were filled with the value of the corresponding available biological duplicate. This was justified by the high similarity between biological replicates at all development stages (r between 0.88 and 0.97, *p* < 0.001, Spearman correlation) (Figure S2) and by their clustering together in principal component analysis (PCA). Venn diagrams were generated in R to visualize overlaps between groups of proteins. To account for missing values attributable to low protein abundance, MinProb imputation [35] was applied to retain proteins with quantified abundance values in at least one developmental stage (Supplementary Table S1). MinProb is a low-tail imputation method that estimates missing values based on the lower tail of the data distribution across developmental stages, which reduces bias and variance in downstream analysis by retaining proteins not detected in one or more developmental stages [8]. See Supplementary spreadsheet S1 for the data corrected for missing duplicates and imputation. Following corrections, the data were subjected to heatmap [36] and bootstrap analysis. Protein abundance values were normalized using z-score transformation and clustered by hierarchical clustering (hclust) in R using Ward’s method [37]. Cluster stability was assessed with bootstrap analysis using the R package pvclust [38].

Differential protein abundance analysis was performed using the limma package in R [39]. Protein abundance values were log_2_-transformed and linear models were fitted to each protein across the five developmental stages. Pairwise comparisons were performed with contrasts defined between adjacent developmental stages to understand stagewise transitions. Overall developmental changes were captured using specific contrasts set to compare abundance at each developmental stage against the average of the remaining stages [10]. Empirical Bayes moderation was applied to improve variance estimation, and *p*-values were adjusted using the Benjamini–Hochberg method to control the FDR. Proteins with a log_2_ fold change (logFC) > 1 were considered proteins with increased abundance, those with a logFC < −1 were considered proteins with decreased abundance and those between −1 and 1 were considered unchanged. Hierarchical clustering was performed on the differential abundance data analyzed for each stage against the average of the other stages to group proteins with similar abundance patterns across developmental stages.

Gene Ontology (GO) functional enrichment analysis was performed using the topGO R package [40] with the “classic” algorithm and Fisher’s exact test, using a GO association file downloaded from the Gene Ontology Consortium (https://current.geneontology.org/products/pages/downloads.html (accessed on 26 February 2025)). Enrichment was evaluated relative to the overall set of proteins identified across all samples. Kyoto Encyclopedia of Genes and Genomes (KEGG) pathway enrichment analysis was performed by comparing the number of observed proteins in each KEGG pathway against the number expected by chance, calculated based on the background distribution of all identified proteins. Statistical significance of enrichment was tested using Fisher’s exact test. Pathway annotations were retrieved from the KEGG database (https://www.kegg.jp/kegg/mapper/search.html (accessed on 7 May 2025)). The enrichment results were visualized in R by plotting the observed-to-expected ratios for each pathway. Expected counts were computed based on the proportion of all background proteins associated with each pathway.

### 2.5. STRING Network Analysis

Differentially abundant proteins clustered using hierarchical clustering were used for network analysis. Protein–protein interaction networks were generated using the STRING database version 12.0 (https://string-db.org/ (accessed on 25 September 2025)) with the organism *D. discoideum*. Networks were generated using the multiple proteins search option. STRING-generated networks were analyzed based on the number of input proteins, total nodes and FDR. The top two local networks with the highest input protein counts and lowest FDR values were selected for detailed analysis. Disconnected nodes were removed and the local networks were further classified into STRING-defined k-means clusters to identify functional subgroups within the network. Final networks were exported from STRING and annotated in Inkscape v1.4.3. Network statistics (number of nodes, edges, average node degree, clustering coefficient and protein–protein interaction enrichment *p*-values) were obtained directly from the STRING output.

## 3. Results and Discussion

### 3.1. General Proteome Statistics and Hierarchical Clustering

**Figure 1:**
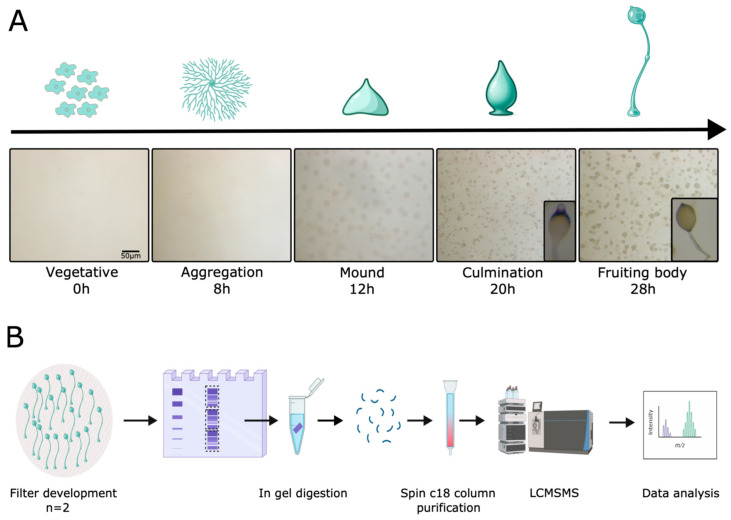
Experimental setup. (**A**) Axenically grown cells were washed with Soerensen buffer and plated on cellulose ester filters to induce multicellular development. Samples were collected at the indicated development stages for a total of 5 proteomics libraries, each in duplicate. Representative images of multicellular development of *D. discoideum* on filters at each development stage. (**B**) Workflow of the study. Created in BioRender. Wollenberg Valero, K. (2026) https://BioRender.com/kjlf7pe.

To achieve highly synchronized and homogeneous development, *D. discoideum* cells were starved and allowed to aggregate, differentiate and complete development on buffered cellulose ester filters (Figure 1A). Samples were harvested immediately after depositing on filters (vegetative stage) and at the aggregation, mound, culmination and mature fruiting body stages for LC-MS/MS (label-free quantification) analysis (Figure 1B).

In total, 4502 proteins were detected and quantified. Based on 12,257 protein-coding genes identified in DictyBase (2 March 2015), the overall rate of recovery is 36.73%. No peptides were detected for 7755 proteins, likely due to low abundance or inherent underrepresentation of particular classes of proteins, like small proteins, membrane proteins, insoluble proteins and those with abundant post-translational modifications [41,42,43,44]. To explore this limitation further, the proportional representation of three classes of proteins was determined that are generally expressed at low to moderate levels and for which comprehensive updated lists have been published: G-protein coupled receptors [45], transcription factors [46] and small GTPase signaling components [47]. As expected, the representation of G-protein coupled receptors was very low: 7 out of 66 (encoded by 69 genes), a 10.61% recovery. A total of 68 transcription factors out of 286 (encoded by 290 genes) were detected, a 23.78% recovery rate below the overall rate. In contrast, the representation of GTPases and their regulators was 42.93% (170 out of 396 proteins encoded by 407 genes), therefore above the overall rate.

**Figure 2:**
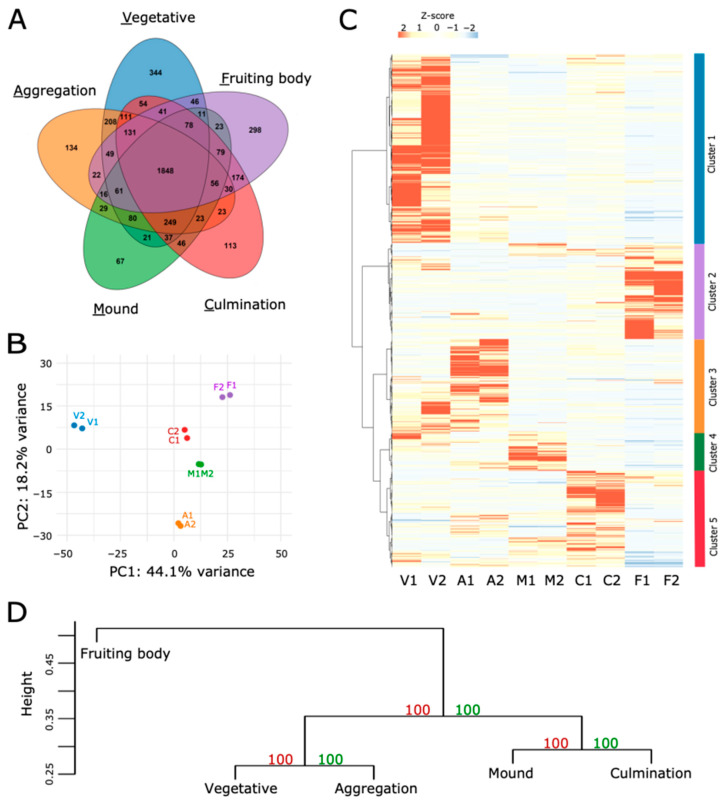
Proteomics analysis of *D. discoideum* showing stage-specific protein abundance across developmental stages. (**A**) Venn diagram illustrating the presence of proteins and their overlap across developmental stages. The analysis was performed before imputation of missing values. (**B**) Principal component analysis (PCA) of protein abundance performed on proteins with abundance values for both biological replicates across all developmental stages. The two replicates for each stage are plotted separately. (**C**) Heatmap of normalized protein abundance for all 4502 proteins across different stages of *D. discoideum* development after imputation of missing values. Abundance values were normalized using z-scores. The z-score color scale is shown in the top left corner. Hierarchical clustering was applied protein (row)-wise using Ward’s method. Five clusters were identified that broadly correspond to predominant protein abundance at one developmental stage. Stages are color-coded in panels (**A**–**C**). (**D**) Hierarchical clustering dendrogram of z-score normalized protein abundances across the five developmental stages. The clustering was performed stage (column)-wise on the same dataset of panel (**C**) using the average of biological replicates. The pvclust R package with correlation distance and average linkage method was used. Bootstrap *p*-values were computed from 1000 resamplings. Approximately unbiased *p*-values (red) and bootstrap probability values (green) are shown at each node.

The data were used to identify proteins exclusively present at one developmental stage or shared between two or more stages (Figure 2A). The analysis revealed a core proteome of 1848 proteins present at all stages. The vegetative stage displayed the highest number of exclusive proteins and the mound stage the lowest (344 and 67, respectively). PCA of the proteomics raw dataset confirmed that variation between the biological replicates was collectively very low and the first principal component showed that the four multicellular stages cluster separately from the vegetative stage (Figure 2B).

For 2654 proteins (approximately 59% of all identified proteins) quantification was missing at one or more time points. To allow differential abundance analysis, missing values were imputed and the resulting dataset was used for all subsequent analyses. Protein abundance values across all developmental stages were normalized using z-score transformation for a heatmap representation and hierarchical clustering (Figure 2C; see Supplementary spreadsheet S2 for the complete dataset), which allowed the identification of five clusters that broadly group proteins of highest abundance at a particular developmental stage. For example, cluster 1 contains proteins predominantly present in the vegetative stage. The analysis of the overall protein abundance patterns by an unsupervised clustering method shows that aggregation forms a cluster with the vegetative stage with the same bootstrap support of a cluster made of mound and culmination stages, and these four stages cluster separately from the fruiting body stage (Figure 2D).

**Figure 3:**
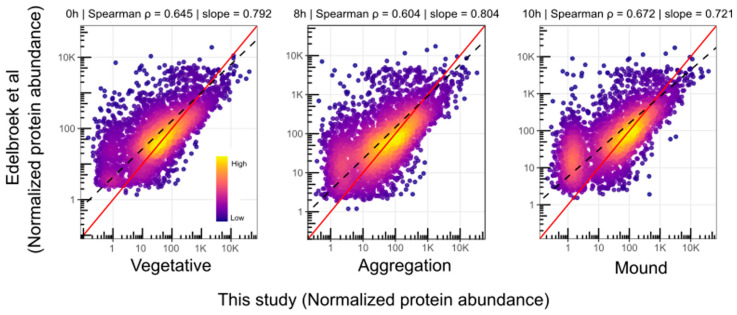
Spearman correlation between protein abundance values for the 0 h, 8 h and 10 h time points of the Edelbroek et al. (2024) study [8] and the vegetative, aggregation and mound stages of this study. Both datasets were converted to linear scale and normalized to counts-per-million by dividing each protein’s abundance value by the sum of all protein abundance values in the corresponding time point or stage, then multiplying by 10^6^. Each dot represents the normalized abundance of one protein quantified in both datasets. Note that the data are plotted in logarithmic scale; however, the correlation was calculated using the linear data. The dashed black line indicates the reduced major axis regression fit, and the red line shows the y = x diagonal. The color scale indicates the density of dots at a given position.

To test the reliability of the proteomics data, comparisons with previously published Western blots and enzyme activity determinations time courses (Figure S3 and Table S2) and with the study by Edelbroek et al. (2024) [8] were undertaken. Spearman correlation showed a low, although significant, correlation coefficient with published Western blot time courses (r = 0.31, *p* = 0.0069) when data were used as published (Figure S4A). This is due to poor overlap of expression patterns of proteins that peak at the early and middle stages of development and can be attributed to variability introduced by the different experimental conditions used to study development across laboratories. When time shifts were introduced in the published data, a high correlation coefficient of the combined data was observed (r = 0.72, *p* = 3.4 × 10^−13^) (Figure S4B). Further validating this study, Spearman correlation with the Edelbroek et al. (2024) [8] proteome at equivalent time points showed high correlation coefficients for each of the three stages analyzed (r between 0.60 and 0.67, *p* < 0.001) (Figure 3).

### 3.2. Pairwise Comparisons of Protein Abundance Between Stages Reveal Sharp Developmental Transitions

**Figure 4:**
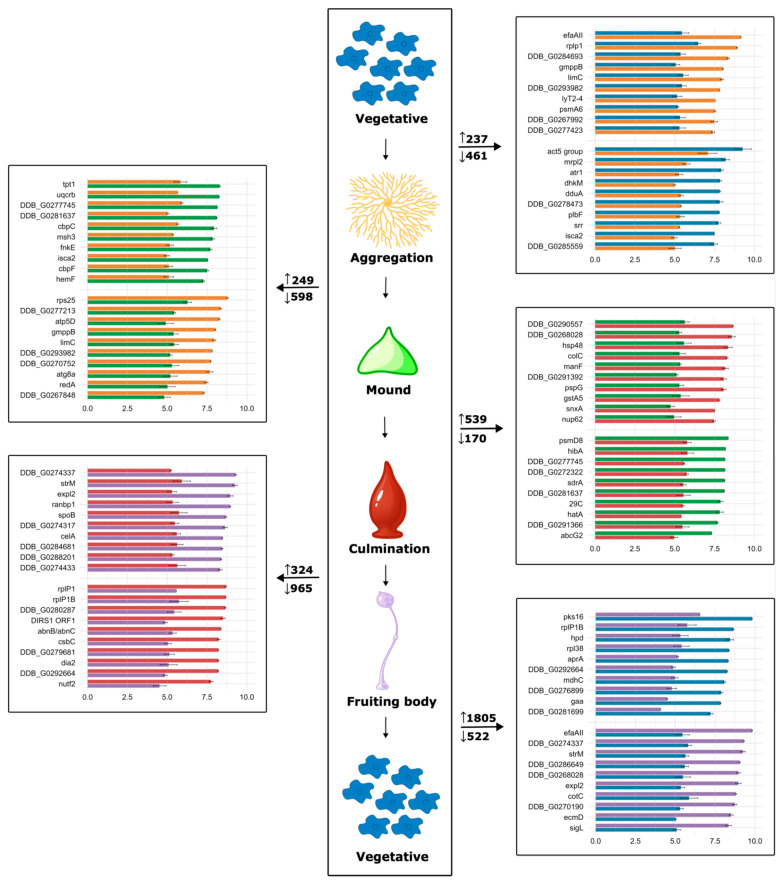
Pairwise comparisons of protein abundance between development stages. For simplicity, gene names are shown. The abundance of each protein at a given stage was compared with the abundance at the immediately following stage. The numbers before each histogram box correspond to the number of differentially abundant proteins (DAPs) with increased (arrow pointing up) or decreased (arrow pointing down) abundance (adjusted *p* < 0.01). The abundance values of the top 10 DAPs with increased or decreased abundance are shown in the top and bottom blocks of histograms, respectively, of each box, and are plotted in log_10_ scale. Bar colors match the colors of the developmental stages. Central panel created in BioRender. Wollenberg Valero, K. (2026) https://BioRender.com/5p4sihy.

A full proteome across the entire *D. discoideum* life cycle presents a unique opportunity to investigate what developmental transitions are accompanied by the most profound changes. For this, the abundance of each protein at a given developmental stage was compared to its abundance at the immediately following stage and expressed as log_2_ of fold change (logFC). These comparisons revealed between 698 and 2327 differentially abundant proteins (DAPs) (adjusted *p* > 0.01) (Table S3). The top 10 proteins with increased (logFC ≥ 1) and decreased (logFC ≤ −1) abundance in each transition are shown in Figure 4 (see Supplementary spreadsheet S3 for the complete analysis). The changes between the vegetative and the aggregation stages and between this and the mound stage represent the transition to multicellularity triggered by starvation [8]. Both were found accompanied by 698 and 847 DAPs, respectively, of which approximately two thirds showed decreased abundance. During formation of the culminant, in which cells continue to differentiate mainly into stalk and spore cells that occupy different positions in the multicellular structure, 709 DAPs were found, about three quarters of them showing increased abundance.

The formation of the mature fruiting body was accompanied by significant changes in 1289 proteins (28.63% of the proteome uncovered in this study). The abundance of approximately three quarters of these DAPs decreased, reflecting the changes that occur during the formation of a stalk composed of dead cells and a sorocarp with compact spores. These observations contrast with the information gained from transcriptomics studies, which have identified starvation, multicellular differentiation and culmination as the transitions accompanied by the major changes, and, apart from the transition from the vegetative to the aggregation stage, the directions of the changes generally do not match [5,6].

The most dramatic change was observed between the fruiting body and the vegetative stage, involving 2327 DAPs (51.68% of the proteome). The abundance of three quarters of these DAPs was found to have increased. This most likely reflects the dramatic changes happening during the transformation of dormant spores into growing amoebae [48] along with the loss of proteins characteristic of specialized structures of the fruiting body, like the stalk and the spore coat. Notably, this transition has not been considered in previous proteomics and transcriptomics studies.

### 3.3. Distinct Patterns of Protein Abundance Across the D. discoideum Life Cycle

While the analysis of protein abundance between developmental stages revealed clear transitions, it does not inform about patterns of protein abundance across the entire life cycle. To address this, the abundance of each protein at a given developmental stage was compared with the average of the abundance at all other stages and expressed as logFC (Supplementary spreadsheet S4). The proteins at each stage were then ranked based on their logFC value and distributed into three categories: increased (logFC ≥ 1), decreased (logFC ≤ −1) and unchanged (logFC between −1.0 and 1.0) abundance. The overall results (Table S4) broadly match the results obtained in the pairwise comparisons. Moreover, the proteins with the highest increase in abundance at a given stage match the most abundant DAPs at the transition with the previous stage.

**Figure 5:**
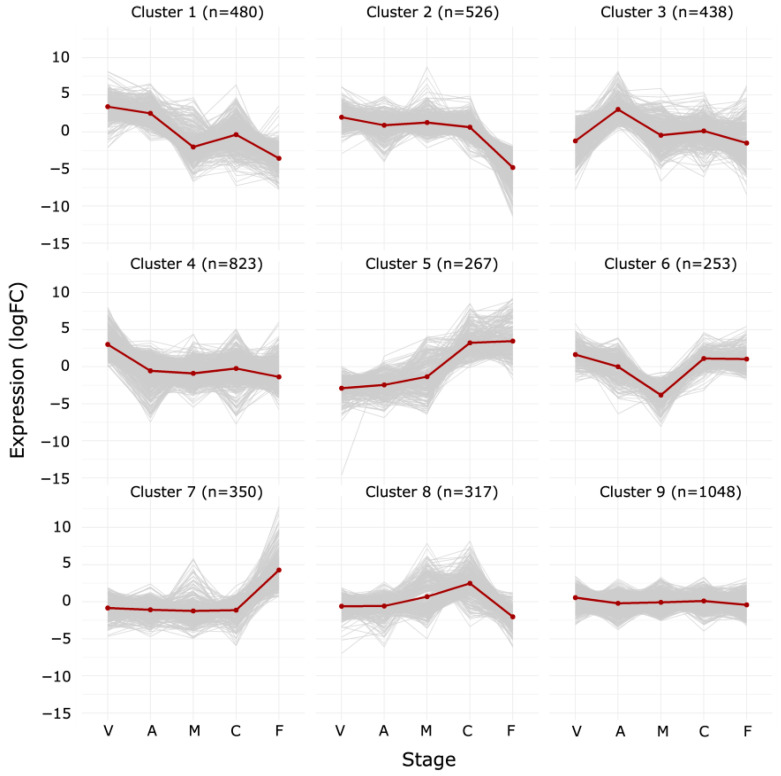
Clustering of differentially abundant proteins across the vegetative (V), aggregation (A), mound (M), culmination (C) and fruiting body (F) stages. Proteins were grouped into nine clusters using hierarchical clustering with Ward’s variance minimization method based on patterns of differential abundance values (log_2_FC) across stages. Gray lines represent individual protein abundance trajectories; the red line indicates the average profile per cluster.

**Figure 6:**
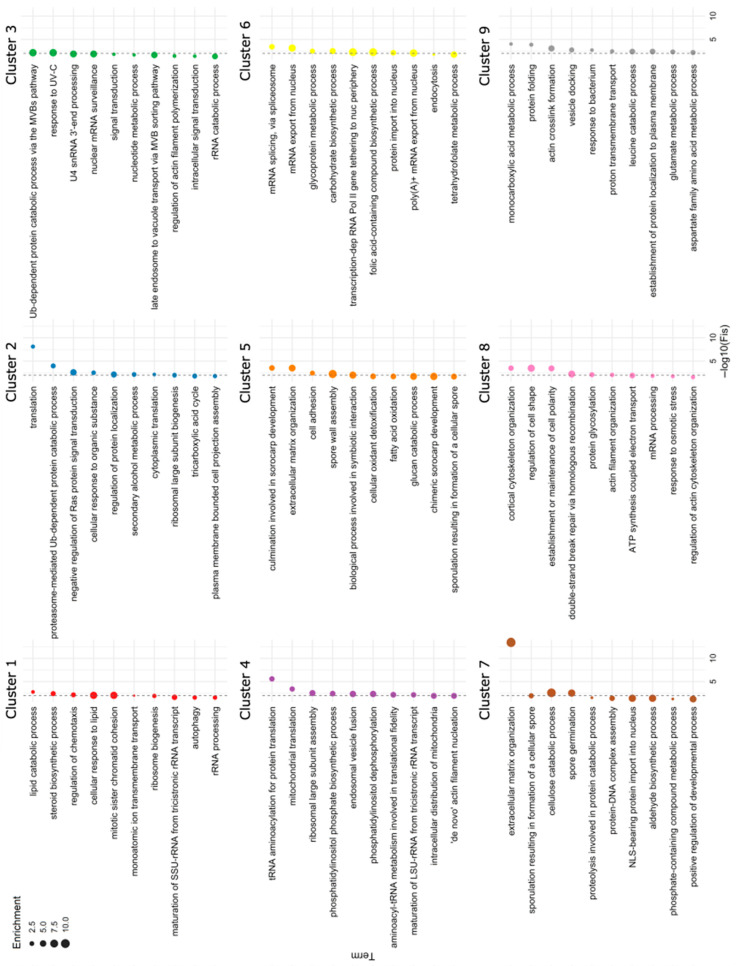
Gene Ontology (GO) biological process enrichment analysis of hierarchical clusters. The ten most significantly enriched GO terms per cluster are shown. *p*-values were calculated using Fisher’s exact test; the vertical dashed line indicates significance at *p* = 0.01 (−log_10_ scale). Bubble size reflects the enrichment score of each GO term.

To assign each protein to a class of proteins with a similar protein abundance profile, hierarchical clustering was applied to the logFC values. Setting the number of clusters to nine for our dataset resulted in distinct patterns of abundance (Figure 5). Tables S5–S12 show the 25 proteins with the highest logFC values at a peak or lowest logFC values at a trough in clusters 1 to 8. The full dataset is presented in Supplementary spreadsheet S5. The ten most significantly enriched GO terms per cluster are shown in Figure 6. We will examine these hierarchical clusters in the context of the changes that occur at early and late stages of the developmental cycle. Cluster 9, the most populous, will be discussed later in the context of the stable proteome.

### 3.4. The Transition to Multicellularity

*D. discoideum* is remarkable for its aggregative mode of development triggered by starvation. The changes in the proteome required for aggregation are captured in clusters 1, 3 and 4.

#### 3.4.1. Cluster 3

The abundance of proteins in cluster 3 peaks at the aggregation stage. Notably, from the 34 proteins in this cluster that have been subjected to functional characterization, at least 15 appear to be required for proper chemotaxis and/or aggregation. Important signaling proteins among them are the RasGEF Aimless, plekstrin homology and Ras domain protein (PHR), RegA, the soluble guanylyl cyclase (sGC) and RacC. Aimless and PHR are components of the Sca1 complex that also includes the RasGEF GefH, the scaffold and catalytic subunits of the protein phosphatase 2A and the scaffold protein Sca1 [49]. The complex is recruited to the leading edge of chemotaxing cells where it regulates the activation of RasC and its downstream target of rapamycin complex 2 (TORC2)-Akt/protein kinase B (PKB) pathway, thereby modulating F-actin dynamics and the relay of chemotactic signals [49]. While Aimless and PHR display a peak of abundance at the aggregation stage, the protein levels of other components of the Sca1 complex do not change relative to the vegetative stage. This suggests that a partial complex may exist in vegetative cells that only becomes fully functional when cells become aggregation competent.

The major intracellular cAMP phosphodiesterase RegA is part of the circuit that generates 6 min oscillations in cAMP during aggregation and includes protein kinase A (PKA), with which it associates, RdeA, ERK2 and DhkA, as well as the cAMP receptor CAR1 and the adenylyl cyclase ACA [1]. Leaving aside CAR1 and ACA, which were not detected in this proteome, RegA is the only component whose abundance peaks at the aggregation stage, suggesting that it constitutes a critical element in the circuit. Consistent with its additional participation in cAMP-regulated processes at later developmental stages, RegA presents a pattern of bimodal protein abundance with a second peak at the culmination stage. sGC is the main source of cyclic GMP (cGMP), important for the localization of myosin and the suppression of pseudopods at the rear of migrating cells [50,51].

RacC is one of several Rho GTPases, along with RacB and Rac1, important for modulating actin remodeling-dependent processes in aggregation competent cells [52]. RacC is important for actin polymerization at the leading edge and ACA localization at the uropod [53,54], but also mediates chemorepulsion [55]. In addition, Rho GTPase regulators important for chemotaxis ElmoA, ElmoE and GacH can be found in cluster 3, along with several uncharacterized Rho GTPase activating proteins and the exchange factor GxcCC. Two actin-binding proteins required for aggregation, the glia maturation factor A [56] and LimC [57] show a specific increase in abundance in the aggregation stage too. Cluster 3 also contains several uncharacterized proteins whose abundance peaks strongly at the aggregation stage, including the a12 subunit of the heterotrimeric G-protein (encoded by *gpaL*) and the small GTPase Rab2B, that may play yet unrecognized roles at early stages of development.

#### 3.4.2. Clusters 1 and 4

Clusters 1 and 4 contain proteins abundant in vegetative cells as opposed to all other stages. Whereas in cluster 4 proteins remain low after the aggregation stage, in cluster 1 the decline is progressive, and some proteins show a secondary peak at the culmination stage. Cluster 4 is the largest after cluster 9 and contains a varied mix of proteins. The most frequent GO terms in this cluster are related to aminoacylation and other modifications of tRNA and to ribosomal subunit processing and assembly, both mitochondrial and cytosolic (Figure 6, Figures S4 and S5). This is consistent with the reported reduced synthesis of rRNA and ribosomal protein genes upon starvation [58] and the observations of an early development proteome study [20]. Intriguingly, kinases and phosphatases involved in phosphatidylinositol metabolism are abundant in this cluster too. Notably, 44% of the top proteins with decreased abundance in cluster 4 are annotated as enzymes, displaying a variety of activities. In cluster 1, ribosomal subunit proteins, proteins involved in rRNA maturation and enzymatic activities (particularly those involved in lipid catabolism and steroid biosynthesis) as well as methyltransferases and RNA helicases are also frequent. Binding to cofactors FAD, FMN and NADP is a feature of many proteins of cluster 1. Regulation of chemotaxis appears as an enriched GO term in this cluster, which can be attributed to the presence of several, mostly uncharacterized, exchange factors and GTPase activating proteins for Ras, Rap and Rac small GTPases probably participating in processes like micropinocytosis and phagocytosis in vegetative cells [59]. Considered collectively, clusters 1 and 4 mainly represent the processes in vegetative cells that need to be shut off when they transition to development.

### 3.5. Culmination and Fruiting Body Maturation

The changes in the proteome after the mound has formed and differentiation and morphogenesis progresses are captured in clusters 2, 5, 6, 7 and 8. These stages are not covered by previous proteomics studies and therefore will be discussed in detail.

#### 3.5.1. Cluster 8

Most proteins in cluster 8 have a peak of abundance at the culmination stage, with approximately one third of the proteins appearing more abundant at the mound stage, or highly abundant at the mound and culmination stages compared to other stages. GO term enrichment analysis reveals a significant accumulation of biological processes related to the cytoskeleton, cell shape and polarity, and molecular functions like calcium ion binding and actin binding (Figure 6), reflecting their importance for the cell responses to morphogenetic signals required for cell sorting in the mound and during culmination. There is a notable accumulation of calcium-binding proteins in this cluster, most of them containing EF-hand domains, including calcium-binding proteins (CBPs) 1, 3, 6, 7, 9, 4a and 4b, the mitochondrial substrate carrier family protein C and the RabGAP encoded by DDB_G0295717. Proteins like CBP1, 3 and 7 seem to play roles during early stages of development and CBP1 and 3 are clearly involved in actin cytoskeleton remodeling [60,61,62]. Two proteins with high affinity for calcium, translationally controlled tumor protein homolog (TCTP) 1 and 2, are found in cluster 8. These proteins are multifunctional and at least TCTP1 regulates aggregate size, prestalk/prespore cell ratio and spore viability [63]. There are also two proteins with a C2 domain (a calcium-dependent phospholipid binding domain): DDB_G0349321 and DDB_G0278101, the latter being described as a putative cell number regulator [64]. Another protein regulated by calcium in this cluster is GRP125, a cytoskeleton protein of the gelsolin family that is indispensable for phototaxis [65].

While most components of the actin cytoskeleton are usually expressed evenly throughout development, several proteins accumulate at the mound and/or culmination stage where they may play specific roles. These include three uncharacterized monomeric actin-binding proteins (actobindin A and profilin-related proteins DDB_G0278017 and DDB_G0280415), the microfilament nucleators formin C and formin D, the fimbrin-related protein FimC, the LIM-domain-containing protein LimD1 and the pH sensor histactophilin II. Notably, formin C is involved in cell–cell adhesion at late stages of development, as its loss results in aberrant sorocarp development [66]. Signaling to the cytoskeleton is represented in cluster 8 by Racs 1, D, J, I and L. Very little is known about RacJ, I and L other than their confirmed predominant expression at RNA level at mid stages of development [67], suggesting specific roles during those stages that might be subtle or overlapping because the knockout of their genes independently did not result in an overt phenotype [68]. Further characterized signaling proteins with an impact on cytoskeleton remodeling and development are the functionally related RacGEF GxcT [68], RasC [69], the Rap1 regulator RapGAPB [70] and the glycogen synthase kinase-like GlkA [71].

#### 3.5.2. Clusters 5 and 7

Clusters 5 and 7 contain proteins with a peak of abundance at the culmination and fruiting body stages or at the fruiting body stage only, respectively. GO term enrichment analysis reveals an accumulation of biological processes related to extracellular matrix organization (particularly in cluster 7), as well as spore coat formation (more prominent in cluster 5) and spore germination (Figure 6). Molecular function GO terms related to carbohydrate binding and cellular component GO terms related to the extracellular region are particularly abundant in these clusters (Figures S5 and S6). This reflects the fundamental roles of extracellular matrix proteins in the final stages of development. As an approximation of their relative frequency, we determined that 34.5% and 26.6% of the proteins in clusters 5 and 7, respectively, feature a predicted signal peptide, compared to just 6.8% in cluster 3. Many of the proteins with a predicted signal peptide in clusters 5 and 7 (40% and 31%, respectively) do not have a known function or carry enzymatic activity, frequently one of a variety of hydrolases (24% and 31%, respectively) (see Supplementary spreadsheet S5 for details).

Among the exported proteins present in clusters 5 and 7, those with carbohydrate-binding domains CBM49 and PA14 are particularly numerous. The CBM49 domain binds to crystalline cellulose [72] and is found in 12 proteins in cluster 5 and in 18 proteins in cluster 7, some of them already annotated as extracellular matrix proteins (encoded by *ecmC*, *D*, *F*, *G* and *L*) and/or directly shown to bind cellulose (encoded by *celB*, *st15*, *stab*, *ecmC* and *ecmD*). These proteins are believed to associate with cellulose in the extracellular matrix deposited on the substratum by migrating slugs but, considering their temporal patterns of accumulation, may play roles in the formation of the stalk and spore walls too. The PA14 domain is found in a family of proteins annotated as a prespore cell-inducing factor, of which seven (PsiD, H, I, K, M, R and DDB_G0293832) were found in cluster 5 and three (PsiA, PsiC and DDB_G020342) in cluster 7. The best-characterized member of this family is PsiA. It induces prespore cell differentiation and might also be involved in prestalk cell differentiation [73].

Among several glycohydrolases identified in clusters 5 and 7, it is worth mentioning cellulases, endoglucanases that specifically cleave internal b-1,4-glucosidic bonds of cellulose. They include cellulase A, two uncharacterized proteins of the same glycohydrolase family 9 in cluster 7 (encoded by *iliG* and *iliH*) and three putative cellulases of the glycohydrolase family 5 in cluster 5 (DDB_G0270190, DDB_G0286025 and DDB_G0286061), the former also carrying a CBM49 domain. Cellulase A is expressed in prespore cells during culmination and also during spore germination, when it is used to digest the cellulose layer of the spore coat to allow hatching of the amoeba [74].

Expansins mediate wall expansion in plants by disrupting non-covalent interactions between cellulose microfibrils and other polysaccharides that tether the microfibrils to one another. We identified three members of a family of expansin-like proteins [75], DdExpL7 and 8 in cluster 5 and DdExpL2 in cluster 5. DdExpL2, whose sequence diverges significantly from that of other expansins, may not function as such; however, its significant accumulation at the final stages of development suggests important roles during maturation of the fruiting body. DdExpL7 is expressed in pstAB cells and stalk and its ablation does not cause a phenotypic change, suggesting redundancy among expansin-like proteins [76].

The analysis recovered five spore coat proteins in cluster 5, the major components SP70 (*cotB*), SP60 (*cotC*) and SP85 (*pspB*), an uncharacterized protein (DDB_G0291392) related to SP85, and a protein of the inner face of the spore coat, SpiA [77,78]. SP65 (*cotE*) was recovered in cluster 7, matching the reported observation that this component, unlike most of the spore coat proteins, is not synthesized during the slug stage but during culmination and is incorporated late into spore coats [79].

Important adhesion proteins are present in cluster 5. They include three members of the Tiger family (TgrC1, B1 and M1) characterized by an IPT/TIG domain and a C-terminal transmembrane anchor, the glycophosphatidylinositol (GPI)-anchored contact site A protein and two uncharacterized proteins related to the contact site protein B (encoded by *staC* and BBD_G0271178). TgrC1 is the gp150 surface glycoprotein responsible for late EDTA-resistant cell–cell adhesion by heterophilic interaction with TgrB1, facilitating the transition to multicellularity [80]. Incidentally, one more family member, TgrD1, is found in cluster 7. The contact site A protein is the gp80 surface glycoprotein responsible for EDTA-resistant homophilic cell–cell adhesion [81]. Contact site B proteins are typically expressed at early stages, where they mediate EDTA-sensitive adhesion [82], suggesting that the two related proteins in cluster 5 may play specific roles mediating cell–cell adhesion at late stages.

Two key enzymes in terminal differentiation of prespore and prestalk cells were found in cluster 5, PKA (both catalytic and regulatory subunits) and the des-methyl-DIF-1 methyltransferase encoded by *dmtA*. The three proteins have a peak of abundance at the culmination stage that matches their reported enzymatic activity [83,84]. In addition to its role in cAMP gradient sensing and chemotaxis, PKA is critical for the differentiation of prespore cells into mature spores. Instructed by signals from prestalk cells, PKA is activated upon the accumulation of intracellular cAMP. Activated PKA is then required for the function of transcription factors like GBF, SrfA, CudA and StkA [85]. A role for PKA in stalk differentiation by cyclic diguanylate has been proposed recently [86]. The expression of the methyltrasferase encoded by *dmtA* is induced by cAMP released by prespore cells. The enzyme catalyzes the last step in the synthesis of differentiation inducing factor-1 (DIF-1) that occurs mainly in prespore cells and DIF-1 induces stalk differentiation [87].

Among the uncharacterized proteins in clusters 5 and 7, nine proteins (four in cluster 5 and five in cluster 7) were identified containing a tandem of two to four cystathionine b-synthase (CBS) domains and no other recognizable domains. The CBS domain binds to, and might mediate regulation by, adenosine derivatives, metal ions or nucleic acids, it is found in proteins with diverse functions, and numerous hereditary diseases are associated with mutations in its amino acid sequence [88]. Most of the 16 similar proteins present in *D. discoideum* accumulate at late stages of development, where they may play specific yet unknown roles.

#### 3.5.3. Cluster 6

Cluster 6 contains proteins whose abundance on average wanes at early stages of development but recovers at late stages. The GO term enrichment analysis reveals an accumulation of proteins implicated in mRNA splicing and export from the nucleus and in nucleocytoplasmic transport of proteins, including several nuclear pore complex proteins (Figure 6, Figures S5 and S6). Also frequent are proteins of carbohydrate biosynthesis and glycoprotein metabolism pathways. Notable among these is the cellulose synthase encoded by *dcsA*, responsible for the cellulose found in the extracellular matrix of the slug sheath and in the walls of stalk and spore cells [89]. Collectively, cluster 6 is consistent with the synthesis and remodeling of extracellular matrix proteins and cell wall components that takes place after the formation of the mound [90,91].

#### 3.5.4. Cluster 2

Cluster 2 contains proteins whose abundance decreases at the fruiting body stage. Proteins in this cluster map to three major biological processes and molecular functions: ribosomal assembly, ubiquitin-dependent protein catabolism and calcium ion binding (Figure 6, Figures S5 and S6). Particularly common in this cluster are components of the ribosome, both eukaryotic and mitochondrial, among them the two proteins in the cluster with the highest decrease in abundance and the ribosomal large subunit proteins L36 and L38, but proteins involved in folding reactions are also found in this cluster. Various proteasome subunits and enzymes of the ubiquitin modification pathway can also be found in cluster 2, as well as at least 15 calcium-binding proteins. In addition, the abundance of numerous cytoskeleton elements and metabolic enzymes was found decreased in the mature fruiting body. These include the actin filament severing protein severin, the calponin homology domain-containing protein DDB0233800, actobindin B and the essential myosin light chain among the top 25 proteins with decreased abundance in the cluster. Among the proteins involved in metabolic processes, the abundance of several components of the mitochondrial electron transport was found to have decreased. These include various cytochromes and subunits of the NADH dehydrogenase (complex I), as well as enzymes involved in diverse metabolic pathways, like D24-sterol methyltransferase required for sterol biosynthesis and glycine cleavage system H-protein 1 required for glycine decarboxylation. These changes likely reflect the protein composition of the mature fruiting body, where the stalk is made of dead vacuolized cells and spores compact their cytoplasm as they mature.

#### 3.5.5. STRING Analysis

**Figure 7:**
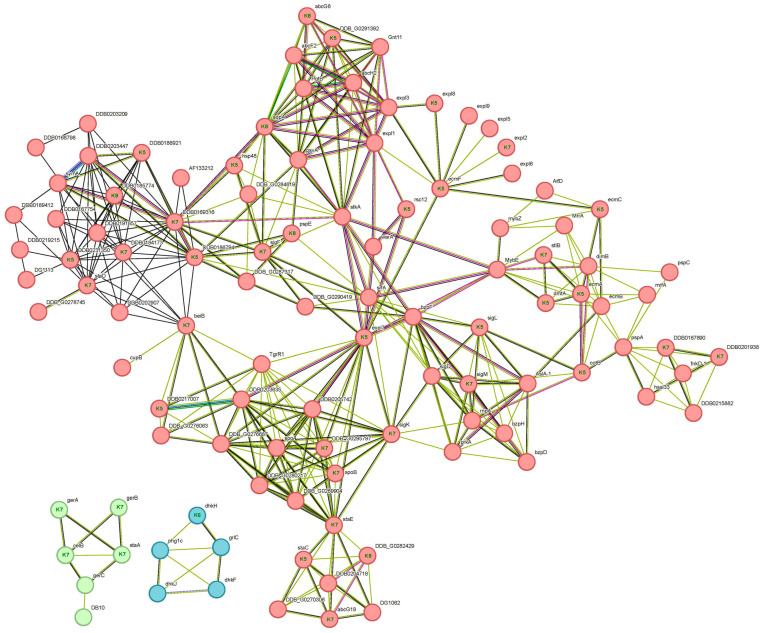
Local network cluster “Anatomical structure morphogenesis/Expansin–pollen allergen/DPBB domain” based on STRING analysis of known and predicted protein–protein interactions generated using hierarchical clusters 5, 6, 7 and 8. The network includes 41 input proteins, labeled inside the nodes with the cluster number to which they belong. Disconnected nodes have been removed. This network was further split into three STRING k-means clusters: mixed group including anatomical structure morphogenesis and Expansin/DPBB domain (red, 95 proteins), spore germination (green, six proteins) and two-component regulatory system and TM9SF proteins (blue, five proteins). Edges (lines) represent functional and physical protein associations, with colors denoting evidence type: curated databases (blue), experimentally determined (pink), gene neighborhood (green), gene fusions (red), co-occurrence (navy), text mining (yellow), co-expression (black) and protein homology (cyan). Network statistics: 106 nodes, 399 edges; FDR, 8.11 × 10^−11^; average node degree 7.25; clustering coefficient 0.704; protein–protein interaction enrichment *p* < 1.0 × 10^−16^.

STRING analysis of clusters 5, 6, 7 and 8 combined identified numerous protein interactions involving 743 proteins from the 1187 that collectively constitute the four clusters (see network details in Supplementary spreadsheet S6). To simplify the analysis, two local network clusters were selected based on highest input protein count and lowest FDR. The “Anatomical structure morphogenesis/Expansin–pollen allergen/DPBB domain” local network cluster (Figure 7) contains 41 input proteins predominantly from clusters 5 and 7. This network cluster contains numerous transcription factors like SrfA, Myb family members MybE and MybZ, MrfA and members of the large bZIP family (BzpD, H and F). SrfA and BzpF both depend on PKA and regulate sporulation [92,93]. MybE is responsible for the differentiation of pstO and anterior-like cells [94] and MrfA regulates the expression of the *ecmA* gene in pstA cells and its absence delays culmination [95].

**Figure 8:**
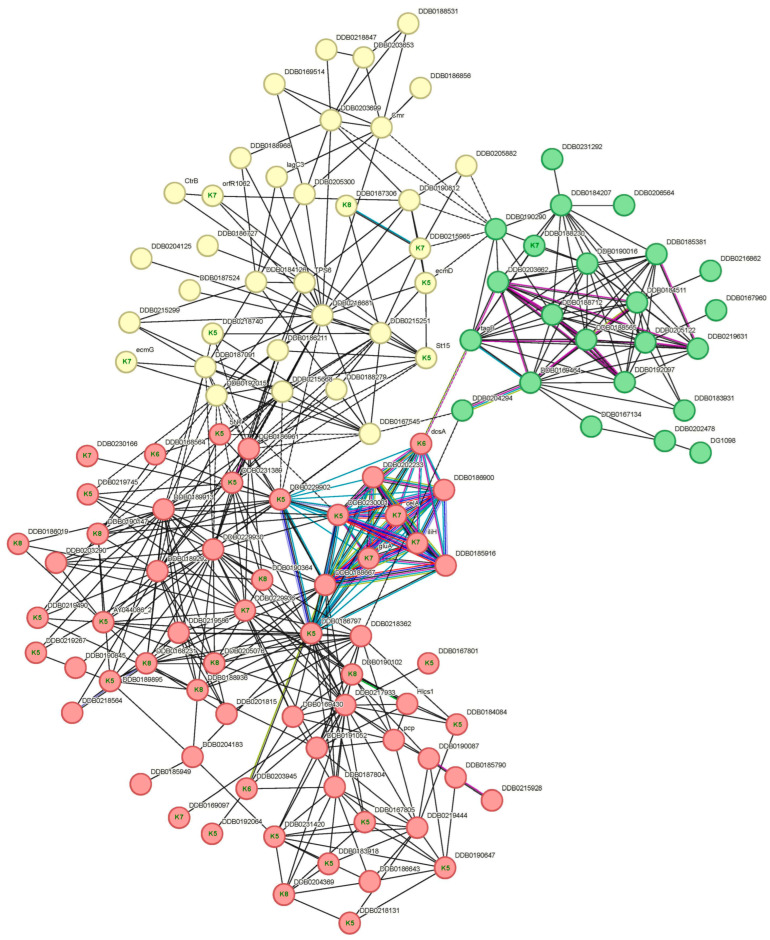
Local network “Hydrolase activity, hydrolyzing O-glycosyl compounds” cluster based on STRING analysis of known and predicted protein–protein interactions generated using hierarchical clusters 5, 6, 7 and 8. The network includes 43 input proteins, labeled inside the nodes with the cluster number to which they belong. Disconnected nodes have been removed. This network was split into three STRING k-means clusters: mostly uncharacterized, including starch and sucrose metabolism proteins (red, 40 proteins), uncharacterized proteins with cellulose-binding/F-box-like function (yellow, 34 proteins) and PLAC8 family and Peptidase S8 subtilisin-related proteins (green, 23 proteins). Edges (lines) represent functional and physical protein associations, with colors denoting evidence type: curated databases (blue), experimentally determined (pink), gene neighborhood (green), gene fusions (red), co-occurrence (navy), text mining (yellow), co-expression (black) and protein homology (cyan). Dotted lines indicate connections between clusters. Network statistics: 190 nodes, 479 edges; FDR, 1.88 × 10^−9^; average node degree 4.98, clustering coefficient 0.431, protein–protein interaction enrichment *p* < 1.0 × 10^−16^.

The “Hydrolase activity, hydrolyzing O-glycosyl compounds” local network cluster (Figure 8) contains 43 proteins, and is subdivided into three connected sub-networks that include proteins with cellulose-binding domains and glycosyl hydrolase activity. Notably, seven proteins are highly connected, the cellulose synthase (encoded by *dcsA*) and six proteins with cellulase activity, encoded by *celA*, *gluA*, *iliH* and the glycohydrolase family 5 proteins encoded by DDB_G0270190, DDB_G0286025 and DDB_G0286061) that have been discussed in Section 3.5.2.

### 3.6. The D. discoideum Continuous Expression and Stable Proteomes

**Figure 9:**
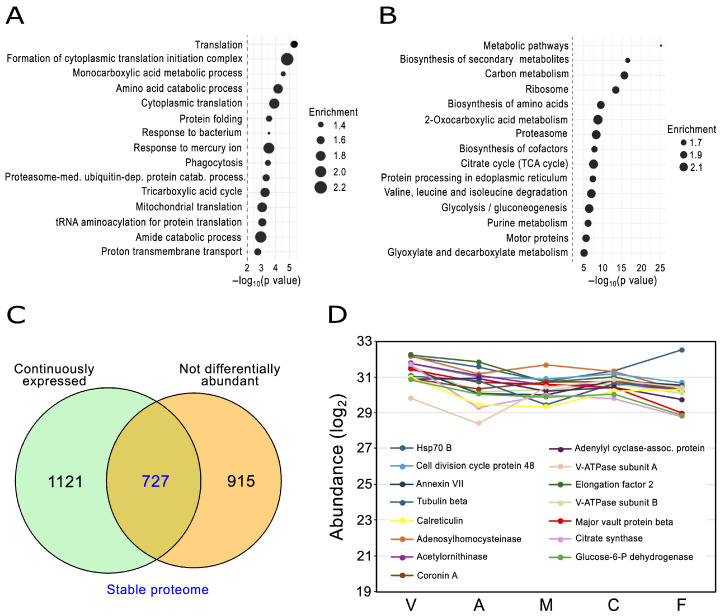
The *D. discoideum* continuous expression and stable proteomes. (**A**) Gene Ontology (GO) biological process enrichment analysis of the continuous expression proteome. See Figure S7 for cellular component and molecular function enrichment analysis. Enrichment analysis was performed using the clusterProfiler package in R. The top 15 most significantly enriched GO terms are shown. Bubble size represents the enrichment score for each GO term. (**B**) KEGG pathway enrichment analysis of the continuous expression proteome. The top 15 most significantly enriched KEGG pathways are shown. Bubble size indicates the enrichment score, calculated as the ratio of observed-to-expected proteins annotated at each pathway. For panels (**A**,**B**), statistical significance was assessed using Fisher’s exact test; a dashed vertical line marks *p* < 0.01 (−log_10_ scale). (**C**) Venn diagram showing the overlap of the continuous expression proteome and a set of not differentially abundant proteins (*p* ≥ 0.01), defining the stable proteome. (**D**) The 15 most abundant proteins in the stable proteome. Abundance values are plotted in log_2_ scale. Y-axis bounds encompass the abundance values from the least to the most abundant proteins in the complete stable proteome. V, vegetative; A, aggregation; M, mound; C, culmination; F, fruiting body.

The Venn diagram in Figure 2A identifies a continuous expression proteome, defined as the set of proteins detected at all stages of development prior to imputation of missing values, of 1848 proteins. It accounts for 41.04% of the proteome described here (Supplementary spreadsheet S7) and is lower than the 68% reported by Edelbroek et al. (2024) [8]; however, this study only extends to the mound stage, whereas the data reported here extend to the profound changes in protein composition that take place during culmination and spore maturation. Continuous expression proteomes reported in other developmental models vary considerably between 59% and 58% in early bovine embryos (4–6 cells to blastocyst) and *D. melanogaster* (egg to adult), respectively, and 14% in zebrafish embryos (4-cell to 5 days post-fertilization) [23,24,96]. To address the functionality of this sizable set of proteins, GO and KEGG pathways enrichment analyses were performed. As expected, the *D. discoideum* continuous expression proteome is enriched for metabolic processes related to energy production and biosynthesis of precursors, particularly monocarboxylic and amino acid metabolism, but also proton and electron transport. Also enriched are protein biosynthesis and processing (folding and degradation), cytoskeleton-related processes, endocytosis and intracellular vesicle trafficking (Figure 9A,B and Figure S7). Those processes are remarkably similar to the ones found in continuous expression proteomes in zebrafish, fruit fly and bovine embryos and reflect the common basic activities of eukaryotic cellular systems [23,24,96].

Having determined the continuous expression proteome, we sought to identify a set of stable proteins, defined as those found in all developmental stages at relatively constant levels. For this, we generated an intersection of the continuous expression proteome with a set of not differentially abundant proteins (those whose logFC does not significantly change based on an adjusted *p* ≥ 0.01) (Figure 9C). The result is a stable proteome of 727 proteins (Supplementary spreadsheet S7). The stable proteome of *D. discoideum* constitutes 39.34% of the continuous expression proteome, very similar to the stable developmental proteome of fruit fly (41.20%) [23]. The stable proteome is enriched in cytoskeleton proteins, including, among the 50 top abundant proteins, tubulins a and b, actin and numerous actin-binding proteins like cortexillin B, coronin A, cyclase associated protein (CAP), actin interacting protein 1 (Aip1), fimbrin, talin B and AbpF. Actin is commonly used as a development loading control [97,98] and tubulin is known to be expressed at constant levels throughout the developmental cycle [99]. Besides cytoskeleton components, heat shock proteins, enzymes involved in various metabolism processes and components of the vacuolar ATPase are among the most abundant proteins in the stable proteome (Figure 9D). Because the hierarchical cluster 9 contains a set of proteins that on average change very little across the developmental cycle, we investigated the composition of this cluster relative to the continuous expression proteome. Nearly all the proteins in cluster 9 are either part of the continuous expression proteome or not differentially abundant, with 538 proteins (51.34% of the cluster) constituting a substantial part of the stable proteome (Figure S8).

## 4. Conclusions

This study is the first to determine the proteome of *D. discoideum* across the complete life cycle, substantially extending previous studies that have focused on early developmental stages [8,20] or on specific cellular components like the slime sheath [16] or the secretome [17,18]. The study revealed clear transitions in the proteome between all stages that are more pronounced between culmination and fruiting body maturation, and between fruiting body and the vegetative stage. It also revealed distinct patterns of protein abundance across the life cycle and a stable proteome enriched in cytoskeleton proteins and metabolic enzymes.

The study nevertheless has limitations, some of which have been mentioned earlier and may explain why only approximately 37% of the 12,257 proteins of the *D. discoideum* proteome were detected. In addition, the proteomics approach used in this study is likely to overlook proteoform diversity resulting from post-translational modifications and alternative splicing events that occur and play specific roles at different stages of development. Studies employing specific enrichment strategies for post-translational modifications or top-down proteomics are expected to yield a more complete picture of the complexity the *D. discoideum* proteome. Although this study was conducted on two independent biological replicates only, robust validation of the data was confirmed by correlation analysis of replicate values at each developmental stage, correlation of the proteome with the data of a recent similar proteomics study conducted under similar conditions (although restricted to early development) and correlation of abundance data of a sample of proteins with published Western blots.

This study is a collection of global proteomes at various stages of development, and consequently cell-type specific detail is not preserved during sample collection. Cell-type specific transcriptomic data are available for prestalk and prespore enriched fractions [100], terminally differentiated cells [10] and multicellular aggregates using single-cell RNA sequencing [9]. It would be interesting to generate proteomes of individual cell types to complement and expand the transcriptomics data available. One more limitation concerns the temporal resolution achieved in this study, which is restricted to relatively long intervals between developmental milestones. A more frequent sampling is likely to provide a more precise understanding of the dynamics of the proteome at the major developmental transitions, similar to the resolution achieved for the transcriptome [5].

Although the proteomes of fruiting body and vegetative stages are compared in this study and dramatic changes in the abundance of many proteins are reported, the process of spore germination, which only lasts for about 3 to 4 h, is not addressed specifically. One study has investigated the transcriptional signature of spore germination [101], and while early studies have documented changes in the proteome using two-dimensional gel electrophoresis [102], large-scale proteomics studies of this process are still missing. Despite these limitations, we believe this study will constitute a valuable resource for future studies addressing a wide range of biological questions related to cell differentiation, development and proteome dynamics in *D. discoideum*, and will serve as a foundation for more targeted, cell-type specific proteomic investigations in the future.

## Data Availability

The proteomics data used in this study have been submitted to the Proteomics Identifications Database (PRIDE) under accession number PXD0620280. All code for downstream analysis of the proteomics datasets can be accessed on figshare at https://doi.org/10.6084/m9.figshare.30620729 (accessed on 14 November 2025) and on GitHub at https://github.com/SarenaBanu/Dictyostelium-discoideum-complete-life-cycle-proteomics.git (accessed on 14 January 2025).

## Supplementary Materials

The following supporting information can be downloaded at https://www.mdpi.com/article/10.3390/proteomes14010003/s1, Supplementary spreadsheet S1: Consolidated raw data, data corrected for missing duplicates and data after imputation. Supplementary spreadsheet S2: Z-scores. Supplementary spreadsheet S3: Pairwise comparisons. Supplementary spreadsheet S4: Comparison of each stage to the rest. Supplementary spreadsheet S5: Hierarchical clusters. Supplementary spreadsheet S6: STRING analysis. Supplementary spreadsheet S7: Continuous expression and stable proteomes. Figure S1: *Dictyostelium discoideum* proteins across the life cycle. Figure S2: Spearman correlation between biological duplicates for each developmental stage; Figure S3: Validation of LC-MS/MS protein abundances using published data; Figure S4: Spearman correlation between LC-MS/MS and published data; Figure S5: Gene Ontology (GO) molecular function enrichment analysis for each hierarchical cluster of differentially abundant proteins during development; Figure S6: Gene Ontology (GO) cellular component enrichment analysis for each hierarchical cluster of differentially abundant proteins during development; Figure S7: The *D. discoideum* continuous expression proteome; Figure S8: Comparison of hierarchical cluster 9 with the continuous expression and stable proteomes; Table S1: Number of proteins where missing replicates were filled and missing values were imputed; Table S2: Summary of proteins validated by comparison of LC-MS/MS protein abundances with published Western blot and enzyme activity data [103,104,105,106,107,108,109]; Table S3: Pairwise comparisons of protein abundance between development stages; Table S4: Differentially abundant proteins at each developmental stage; Table S5: Hierarchical cluster 1 proteins with a steady decline of abundance across the developmental cycle; Table S6: Hierarchical cluster 2 proteins with a trough of abundance at the fruiting body stage; Table S7: Hierarchical cluster 3 proteins with a peak of abundance at the aggregation stage; Table S8: Hierarchical cluster 4 proteins with a peak of abundance at the vegetative stage; Table S9: Hierarchical cluster 5 proteins with a peak of abundance at the culmination and fruiting body stages; Table S10: Hierarchical cluster 6 proteins with a trough of abundance at the mound stage; Table S11: Hierarchical cluster 7 proteins with a peak of abundance at the fruiting body stage only; Table S12: Hierarchical cluster 8 proteins with a peak of abundance at the culmination stage.

## Abbreviations

The following abbreviations are used in this manuscript:

ACA	Adenylyl cyclase A
cAMP	Cyclic adenosine monophosphate
CAP	Cyclase associated protein
cAR1	cAMP receptor 1
CBP	Calcium-binding protein
CBS	Cystathionine b-synthase
cGMP	Cyclic guanidine monophosphate
DAP	Differentially abundant protein
DIF-1	Differentiation inducing factor-1
EDTA	Ethylenediaminetetraacetic acid
FC	Fold change
FDR	False discovery rate
GO	Gene Ontology
KEGG	Kyoto Encyclopedia of Genes and Genomes
LC-MS/MS	Liquid chromatography–tandem mass spectrometry
LFQ	Label-free quantification
PCA	Principal component analysis
PHR	Plekstrin homology and Ras domain protein
PKA	Protein kinase A
PKB	Protein kinase B
sGC	Soluble guanylyl cyclase
TCTP	Tumor protein homolog
TORC2	Target of rapamycin complex 2
