# Correction: Banu et al. The Proteome of *Dictyostelium discoideum* Across Its Entire Life Cycle Reveals Sharp Transitions Between Developmental Stages. *Proteomes* 2026, *14*, 3

**DOI:** 10.3390/proteomes14020018

**Published:** 2026-04-21

**Authors:** Sarena Banu, P. V. Anusha, Pedro Beltran-Alvarez, Mohammed M. Idris, Katharina C. Wollenberg Valero, Francisco Rivero

**Affiliations:** 1 Centre for Biomedicine, Hull York Medical School, Faculty of Health Sciences, University of Hull, Hull HU6 7RX, UK; s.nagoor-pitchai-2021@hull.ac.uk (S.B.); pedro.beltran-alvarez@hyms.ac.uk (P.B.-A.); 2 Energy and Environment Institute, University of Hull, Hull HU6 7RX, UK; 3 CSIR-Centre for Cellular and Molecular Biology, Uppal Road, Hyderabad 500007, Telangana, India; anusha.ccmb@csir.res.in (P.V.A.); idris.ccmb@csir.res.in (M.M.I.); 4 School of Biology and Environmental Science, University College Dublin, D04 V1W8 Dublin, Ireland; katharina.wollenbergvalero@ucd.ie; 5 Conway Institute, University College Dublin, D04 V1W8 Dublin, Ireland

In the original publication [1], the gel image was missing. The gel image has been added to the supplementary material as Figure S1 and the citation has been added to the first paragraph of section 2.2 and Supplementary Materials Section:

A total of 100 µg of protein was mixed with 5× loading dye (10% SDS, 500 mM DTT, 50% glycerol, 250 mM Tris-HCl, 0.5% bromophenol blue, pH 6.8), heated at 95 °C for 5 min and resolved per lane on a 12% sodium dodecyl sulfate-polyacrylamide gel and stained with Coomassie blue (Figure S1). To avoid cross-lane mixing, empty lanes were left between samples, and each developmental stage was run on a separate gel (Figure S1).

**Supplementary Materials**: The following supporting information can be downloaded at https://www.mdpi.com/article/10.3390/proteomes14010003/s1, Supplementary spreadsheet S1: Consolidated raw data, data corrected for missing duplicates and data after imputation. Supplementary spreadsheet S2: Z-scores. Supplementary spreadsheet S3: Pairwise comparisons. Supplementary spreadsheet S4: Comparison of each stage to the rest. Supplementary spreadsheet S5: Hierarchical clusters. Supplementary spreadsheet S6: STRING analysis. Supplementary spreadsheet S7: Continuous expression and stable proteomes. Figure S1: *Dictyostelium discoideum* proteins across the life cycle. Figure S2: Spearman correlation between biological duplicates for each developmental stage; Figure S3: Validation of LC-MS/MS protein abundances using published data; Figure S4: Spearman correlation between LC-MS/MS and published data; Figure S5: Gene Ontology (GO) molecular function enrichment analysis for each hierarchical cluster of differentially abundant proteins during development; Figure S6: Gene Ontology (GO) cellular component enrichment analysis for each hierarchical cluster of differentially abundant proteins during development; Figure S7: The *D. discoideum* continuous expression proteome; Figure S8: Comparison of hierarchical cluster 9 with the continuous expression and stable proteomes; Table S1: Number of proteins where missing replicates were filled and missing values were imputed; Table S2: Summary of proteins validated by comparison of LC-MS/MS protein abundances with published Western blot and enzyme activity data [103–109]; Table S3: Pairwise comparisons of protein abundance between development stages; Table S4: Differentially abundant proteins at each developmental stage; Table S5: Hierarchical cluster 1 proteins with a steady decline of abundance across the developmental cycle; Table S6: Hierarchical cluster 2 proteins with a trough of abundance at the fruiting body stage; Table S7: Hierarchical cluster 3 proteins with a peak of abundance at the aggregation stage; Table S8: Hierarchical cluster 4 proteins with a peak of abundance at the vegetative stage; Table S9: Hierarchical cluster 5 proteins with a peak of abundance at the culmination and fruiting body stages; Table S10: Hierarchical cluster 6 proteins with a trough of abundance at the mound stage; Table S11: Hierarchical cluster 7 proteins with a peak of abundance at the fruiting body stage only; Table S12: Hierarchical cluster 8 proteins with a peak of abundance at the culmination stage.

The figure numbers in other supplementary materials and their citations in the text have also been updated accordingly.

**Figure S1:**
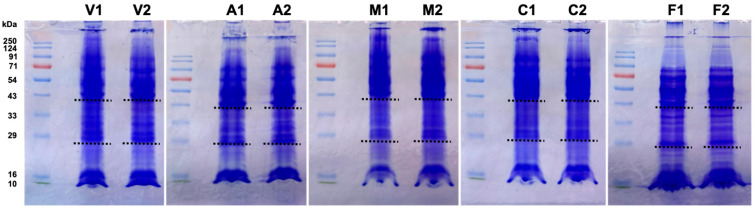
*Dictyostelium discoideum* proteins across the life cycle. Samples (100 μg) were resolved on 12% SDS–PAGE gels and stained with Coomassie blue. Vegetative, aggregation, mound, culmination and fruiting body stages are indicated as V, A, M, C and F, respectively. Biological replicates are denoted as 1 and 2. Black dotted lines indicate the gel lane fractions (high, medium and low molecular weight) excised for in-gel digestion.

Details were missing from Sections 2.2, paragraph 1 and should read as follows:

Vegetative cells (0 h) were collected immediately after depositing on filters, and aggregation (8 h), mound (12 h), culmination (20 h) and fruiting body (28 h) stages were collected based on developmental morphology. Biological replicates were collected from two experiments performed on different days. Cells were scraped off the filters, washed twice with Soerensen buffer, immediately lysed using homogenization buffer (7 M urea, 2 M thiourea, 18 mM Tris HCl, 4% CHAPS, 14 mM Trizma base, 0.2% Triton X-100, 50 mM dithiothreitol and Complete™ ethylenediaminetetraacetic acid (EDTA)-free protease inhibitor cocktail (Roche Diagnostics GmbH, Mannheim, Germany), pH 7.4), snap-frozen and stored at −80 °C. Samples were later thawed and centrifuged at 20,000× *g* for 30 min at 4 °C to separate cell debris [29,30]. Supernatants were collected and proteins quantified using the Amido Black assay [31]. A total of 100 µg of protein was mixed with 5× loading dye (10% SDS, 500 mM DTT, 50% glycerol, 250 mM Tris-HCl, 0.5% bromophenol blue, pH 6.8), heated at 95 °C for 5 min and resolved per lane on a 12% sodium dodecyl sulfate-polyacrylamide gel and stained with Coomassie blue (Figure S1). To avoid cross-lane mixing, empty lanes were left between samples, and each developmental stage was run on a separate gel (Figure S1). Each lane was sliced with a sterile razor blade into three fractions (high, medium and low molecular weight) to improve peptide recovery and coverage across different protein sizes. Fractions were minced into small pieces and transferred to low-binding microcentrifuge tubes. Gel pieces were destained with destaining solution (50% acetonitrile and 40 mM ammonium bicarbonate), and then washed with nuclease-free water followed by 100% acetonitrile and digested with 400 ng of Trypsin Gold (10 ng/µL) (Promega, Southampton, UK) overnight at 37 °C following the manufacturer’s instructions [32]. The three fractions of each sample were processed separately and their MS results subsequently combined.

Details were missing from Section 2.3 and should read as follows:

Raw MS data were analyzed using Sequest HT in Proteome Discoverer™ 2.2.3 (ThermoFisher Scientific, Waltham, MA, USA) against the *D. discoideum* database downloaded from NCBI in April 2024. Label-free quantification (LFQ) was performed using the Minora Feature Detector node integrated in Proteome Discoverer™ 2.2.3 (ThermoFisher Scientific, Waltham, MA, USA), which detects chromatographic features across all runs, aligns them based on retention time and mass-to-charge ratio (*m*/*z*) and quantifies precursor ion intensities. Protein abundance was calculated by adding up the normalized intensities of all unique peptides assigned to each protein group. The Precursor Ion Quantifier node was used for normalization across samples to correct for injection and instrument variability. The false discovery rate (FDR) and XCorr (score vs. charge) thresholds were set at 1%. A minimum of one peptide was sufficient for canonical protein identification. All protein IDs were initially mapped to gene IDs using DictyBase. For mapped proteins lacking a gene ID, BLAST+ (v2.16.0) searches were performed against the NCBI non-redundant database restricted to *D. discoideum*. Duplicate protein entries were consolidated under single gene IDs and their peptide abundances added up. Identical proteins encoded by retrotransposable elements DIRS1 and TRE5 that mapped to different gene IDs were manually consolidated under single representative gene IDs and their peptide abundances added up. The consolidated raw data can be found in Supplementary spreadsheet S1.

Reference to Figure S6 has been added in Section 3.5.2., paragraph 1 and should read as follows:

Clusters 5 and 7 contain proteins with a peak of abundance at the culmination and fruiting body stages or at the fruiting body stage only, respectively. GO term enrichment analysis reveals an accumulation of biological processes related to extracellular matrix organization (particularly in cluster 7), as well as spore coat formation (more prominent in cluster 5) and spore germination (Figure 6). Molecular function GO terms related to carbohydrate binding and cellular component GO terms related to the extracellular region are particularly abundant in these clusters (Figures S5 and S6). This reflects the fundamental roles of extracellular matrix proteins in the final stages of development. As an approximation of their relative frequency, we determined that 34.5% and 26.6% of the proteins in clusters 5 and 7, respectively, feature a predicted signal peptide, compared to just 6.8% in cluster 3. Many of the proteins with a predicted signal peptide in clusters 5 and 7 (40% and 31%, respectively) do not have a known function or carry enzymatic activity, frequently one of a variety of hydrolases (24% and 31%, respectively) (see Supplementary spreadsheet S5 for details).

The authors state that the scientific conclusions are unaffected. This correction was approved by the Academic Editor. The original publication has also been updated.

## References

[1] Banu S., Anusha P.V., Beltran-Alvarez P., Idris M.M., Wollenberg Valero K.C., Rivero F. (2026). The Proteome of *Dictyostelium discoideum* Across Its Entire Life Cycle Reveals Sharp Transitions Between Developmental Stages. Proteomes.

