# Supplementary material for: The Proteome of Dictyostelium discoideum Across Its Entire Life Cycle Reveals Sharp Transitions Between Developmental Stages
Source: Proteomes. 2026 Jan 8;14(1):3. doi: 10.3390/proteomes14010003 (PMC12821622; doi:10.3390/proteomes14010003)
Supplement: Supplementary file 1 [file proteomes-14-00003-s001.zip › Banu et al Proteomes Supplementary.pdf]

Supplementary material

Supplementary tables

Table S1. Number of proteins where missing replicates were filled and missing values were imputed. Note that filling of missing duplicates and imputation may affect the same protein at more than one stage. Percentages are relative to the total number of proteins (4502) identified in the study. V, vegetative; A, aggregation; M, mound; C, culmination; F, fruiting body.

Stages →	V	A	M	C	F
Number of proteins with biological duplicates filled	294	202	104	153	645
Percentage of filled duplicates	6.53	4.48	2.31	3.39	14.32
Number of proteins with imputed values	1133	1432	1778	1409	1539
Percentage of imputed proteins	25.16	31.80	39.49	31.29	34.18

Table S2. Summary of proteins validated by comparison of LC–MS/MS protein abundances with published western blot and enzyme activity data. The enzyme activities collected were: dichloro-THPH methyltransferase (DmtA), protein kinase A (catalytic), protein kinase A (regulatory) and UDP-glucose pyrophosphorylase (UDPGP).

Protein name	Gene name	Development conditions	Reference	Figure number in publication
Contact site A	*csaA*	Phosphate agar	[98]	Fig. 5C
Cystatin A1	*cpiA*	Filter development	[103]	Fig. 3B
Cystatin A2	*cpiB*	Filter development	[103]	Fig. 3B
DmtA (Dichloro-THPH methyltransferase)	*dmtA*	KK2 agar	[84]	Fig. 6
DdDCX (Doublecortin)	*dcx*	Phosphate agar?	[104]	Fig. 2A
Filamin interacting protein (FIP)	*fip*	Phosphate agar	[105]	Fig. 2B
Gelsolin-related protein 125 (GRP125)	*gnrA*	Filter development	[65]	Fig. 6
Interaptin	*abpD*	Filter development	[106]	Fig. 4B
Protein kinase A, catalytic (PKAc)	*pkaC*	Phosphate agar	[83]	Fig. 3
Protein kinase A, regulatory (PKAr)	*pkaR*	Phosphate agar	[83]	Fig. 3
RegA	*regA*	KK2 agar	[107]	Fig. 1
SP85	*pspB*	Filter development	[79]	Fig. 7
SpiA	*spiA*	Filter development	[79]	Fig. 7
TgrC1	*tgrC1*	Filter development	[108]	Fig. 2B
UDPGP (UDP-glucose pyrophosphorylase)	*uppA* and *ugpB*	Filter development	[109]	Fig. 4

Table S3. Pairwise comparisons of protein abundance between development stages. The abundance of each protein at a given stage was compared with the abundance at the immediately following stage and expressed as the log_2 fold change (logFC). Numbers of proteins with logFC ≥ 1, logFC ≤ -1 or between 1 and -1 are shown. Percentages are relative to the 4502 proteins identified. Adjusted p values were determined using the Benjamini-Hochberg method with the limma differential expression package and differentially abundant proteins (DAPs) were considered at adjusted $p < 0.01$. V, vegetative; A, aggregation; M, mound; C, culmination; F, fruiting body.

Transition	Proteins with increased abundance (logFC≥1)	DAPs with increased abundance	Proteins with decreased abundance (logFC≤-1)	DAPs with decreased abundance	Proteins with unchanged abundance (-1<logFC<1)	Total DAPs
**A vs V**	876	237	1878	461	1748	698 (15.50%)
**M vs A**	837	249	1823	598	1842	847 (18.81%)
**C vs M**	1810	539	697	170	1995	709 (15.74%)
**F vs C**	768	324	2362	965	1372	1289 (28.63%)
**V vs F**	2710	1805	923	522	869	2327 (51.68%)

Table S4. Differentially abundant proteins at each developmental stage. Proteins were ranked according to their log_2 fold change (logFC) of abundance at the indicated stage relative to the average of all other stages. The numbers of proteins with increased (logFC ≥ 1), decreased (logFC ≤ -1) and unchanged ($-1 < \text{logFC} < 1$) abundance and their proportion to the 4502 proteins identified were calculated. 197 proteins were found unchanged across all stages.

Stage	Increased abundance	Decreased abundance	Unchanged abundance
**Vegetative**	2357 (52.35%)	835 (18.54%)	1310 (29.09%)
**Aggregation**	1455 (32.31%)	1057 (23.47%)	1990 (44.20%)
**Mound**	834 (18.52%)	1847 (41.02%)	1821 (40.44%)
**Culmination**	1381 (30.67%)	805 (17.88%)	2316 (51.44%)
**Fruiting body**	940 (20.87%)	2424 (53.84%)	1138 (25.27%)

Table S5. Hierarchical cluster 1 proteins with a steady decline of abundance across the life cycle. The 25 top proteins have been ranked according to their $\log_2$ fold change (LogFC) of abundance at the vegetative stage relative to all other stages pooled. Information was retrieved from dictyBase. Protein functions, when not determined experimentally, are deduced from homologous proteins in other organisms or from their domain architecture.

LogFC	Gene ID	Gene name	Protein	Function
8.1341	DDB_G0277213		Similar to mitochondrial ATP synthase subunit d	Unknown
8.1019	DDB_G0293904	*redA*	NADPH-cytochrome-P450 oxidoreductase	Oxidation-reduction processes
7.7366	DDB_G0283871		Uncharacterized protein	Unknown
7.3375	DDB_G0275069	*pks16*	Putative fatty acid synthase 16	Synthesis of fatty acids
7.2411	DDB_G0269900	*gnt10*	GlcNAc transferase	Protein glycosylation
6.4121	DDB_G0291394		Fungal lipase-like domain-containing protein	Lipid hydrolysis in the extracellular space
6.3846	DDB_G0282133		Uncharacterized protein	Unknown
6.1757	DDB_G0276413		Armadillo repeat-containing protein	Unknown
6.1537	DDB_G0269016	*cyp524A1*	Cytochrome P450 family protein	Oxidation-reduction processes
5.9684	DDB_G0286513		Rab GTPase domain-containing protein	Intracellular vesicle transport
5.9616	DDB_G0268008		FAD dependent oxidoreductase domain-containing protein	Oxidation-reduction processes
5.9503	DDB_G0285505	*ino1*	Inositol-3-phosphate synthase	Phospholipid biosynthesis
5.9307	DDB_G0275751		Translation initiation factor IF-2	Protein biosynthesis
5.9302	DDB_G0283793		PKC conserved region 2 containing protein	Unknown
5.9081	DDB_G0284055		Ankyrin repeat-containing protein	Unknown
5.8310	DDB_G0292644		Uncharacterized protein	Unknown
5.7756	DDB_G0281469	*rpl29*	60S ribosomal protein L29	Structural constituent of the ribosome
5.7523	DDB_G0295715		NOL1/NOP2/Sun family protein	tRNA methylation
5.7163	DDB_G0281393	*cxdA*	Cytochrome c oxidase subunit IV, mitochondrial	Oxidation-reduction processes
5.6924	DDB_G0278525	*plaA*	Phospholipase A2	Lipid metabolism
5.6621	DDB_G0288529		POTE ankyrin domain family member A	Unknown
5.6276	DDB_G0280021	*mcfL*	Mitochondrial substrate carrier family protein L	Transmembrane transport
5.6007	DDB_G0291029		Sulfate adenylyltransferase	Sulfur amino acid metabolism
5.5793	DDB_G0269550	*samm50*	Sorting and assembly machinery component 50 like protein	Mitochondrial outer membrane sorting
5.5727	DDB_G0269860		Putative importin 7	Nuclear protein import

Table S6. Hierarchical cluster 2 proteins with a trough of abundance at the fruiting body stage. The 25 top proteins have been ranked according to their $\log_2$ fold change (LogFC) of abundance at the fruiting body stage relative to all other stages pooled. Information was retrieved from dictyBase. Protein functions, when not determined experimentally, are deduced from homologous proteins in other organisms or from their domain architecture.

LogFC	Gene ID	Gene name	Protein	Function
-11.3865	DDB_G0292664		Calponin-homology (CH) domain-containing protein	Actin cytoskeleton remodeling
-11.0843	DDB_G0287995	*rplp1B*	60S acidic ribosomal protein P1β	Structural component of the ribosome
-10.8962	DDB_G0276425	*nutf2*	Nuclear transport factor 2 (NTF-2)	Protein transport into the nucleus
-10.4957	DDB_G0268302	*rpl38*	S60 ribosomal protein L38	Structural component of the ribosome
-10.2350	DDB_G0291221		DIRS1 ORF1	Retrotransposable element
-9.9189	DDB_G0269520	*abnB; abnC*	Actobindin B and C	Actin cytoskeleton remodeling
-9.8740	DDB_G0272756	*csbC*	Contact site B protein C	Cell-cell adhesion during early development
-9.7993	DDB_G0289327	*sevA*	Severin	Actin cytoskeleton remodeling
-9.7967	DDB_G0277511	*hpd*	4-hydroxyphenylpyruvate dioxygenase	Aromatic amino acid metabolism
-9.7497	DDB_G0269790	*gaa*	α-glucosidase	Glycogen metabolism
-9.7383	DDB_G0277859	*mlcE*	Essential myosin light chain	Structural support and enzymatic activity of myosin
-9.4644	DDB_G0268042		CS domain-containing protein	Unknown
-9.4345	DDB_G0287773	*gcvH1*	Glycine cleavage system H-protein 1	Glycine decarboxylation via glycine cleavage system
-9.4023	DDB_G0287811	*cpn10A*	Chaperonin Cpn10 family protein	Protein folding
-9.3338	DDB_G0291091	*mapbpip*	Putative mitogen-activated protein binding protein interacting protein	Late endosome function
-9.0700	DDB_G0290783	*rabG2*	Rab protein G2	Regulation of vesicle trafficking
-8.8777	DDB_G0291362	*rpc19*	DNA-directed RNA polymerases I and III subunit rpc19	
-8.7369	DDB_G0272394	*ndufa5*	NADH dehydrogenase [ubiquinone] 1α subcomplex subunit 5	Mitochondrial electron transport
-8.6245	DDB_G0281699		28S ribosomal protein S29, mitochondrial	Structural component of the ribosome (mitochondrial)
-8.5854	DDB_G0272969	*psmB1*	20S proteasome subunit β-1	Structural component of the proteasome
-8.5482	DDB_G0279681		Calcium-binding EF-hand domain-containing protein	Unknown
-8.4318	DDB_G0285783	*ndufa12*	NADH dehydrogenase [ubiquinone] 1 α subcomplex subunit 12	Mitochondrial electron transport
-8.3629	DDB_G0281093	*rpl37A*	60S ribosomal protein L37a	Structural component of the ribosome
-8.2737	DDB_G0270356	*erg2*	C-8 sterol isomerase	Ergosterol biosynthesis
-8.2374	DDB_G0285489		TRM112 family protein	Peptide and RNA methylation

Table S7. Hierarchical cluster 3 proteins with a peak of abundance at the aggregation stage. The 25 top proteins have been ranked according to their log_2 fold change (LogFC) of abundance at the aggregation stage relative to all other stages pooled. Information was retrieved from dictyBase. Protein functions, when not determined experimentally, are deduced from homologous proteins in other organisms or from their domain architecture.

LogFC	Gene ID	Gene name	Protein	Function
8.1724	DDB_G0269038	*atp5d*	ATP synthase F1δ	ATP synthesis coupled proton transport
7.9127	DDB_G0282969	*masB*	Malate synthase	Glyoxylate cycle
7.8238	DDB_G0293982		Uncharacterized protein	Unknown
7.4485	DDB_G0286191	*atg8a*	Autophagy protein 8a	Autophagy
7.2829	DDB_G0288449	*rpc2*	RNA polymerase III, second largest subunit	Transcription of DNA into RNA (5S and tRNA)
7.1520	DDB_G0290547		Ubiquitin-conjugating enzyme E2 4	Protein degradation
6.9224	DDB_G0281585	*cpsf1*	CPSF domain-containing protein	mRNA polyadenylation
6.6919	DDB_G0287619	*gmppB*	Mannose-1-phosphate guanylyltransferase β	GDP-mannose biosynthesis
6.6812	DDB_G0282595	*gmfA*	ADF-H domain-containing protein	Actin cytoskeleton remodeling
6.5638	DDB_G0277423		Uncharacterized protein	Unknown
6.5157	DDB_G0277881	*limC*	LIM-type zinc finger-containing protein C	Actin cytoskeleton remodeling
6.4377	DDB_G0278907	*mobA*	Mps1 binder-like protein MobA	Unknown
6.2885	DDB_G0272266	*ndufa9*	NADH dehydrogenase [ubiquinone] 1a subcomplex subunit 9	Respiratory chain
6.2609	DDB_G0280351	*gpaL*	G-protein subunit α12	Signal transduction
6.1368	DDB_G0291520	*iliO*	Putative RNA ligase	Unknown
6.0426	DDB_G0267992		Dual specificity protein phosphatase	Unknown
6.0071	DDB_G0272138	*rab2B*	Rab GTPase 2B	Intracellular membrane trafficking
5.7552	DDB_G0280513		RING domain-containing protein	Protein ubiquitination
5.6521	DDB_G0290027		bolA family protein	Unknown
5.5651	DDB_G0283843		Uncharacterized protein	Unknown
5.4943	DDB_G0291155		RNA-binding region RNP-1 domain-containing protein	Unknown
5.4620	DDB_G0268452		v-SNARE family protein	Intracellular vesicle-mediated transport
5.4245	DDB_G0277907	*rplp1*	60S acidic ribosomal protein P1	Structural constituent of the ribosome
5.3000	DDB_G0275161		Uncharacterized protein	Unknown
5.2889	DDB_G0272152		Uncharacterized protein	Unknown

Table S8. Hierarchical cluster 4 proteins with a peak of abundance at the vegetative stage. The 25 top proteins have been ranked according to their $\log_2$ fold change (LogFC) of abundance at the mound stage relative to all other stages pooled. Information was retrieved from dictyBase. Protein functions, when not determined experimentally, are deduced from homologous proteins in other organisms or from their domain architecture.

LogFC	Gene ID	Gene name	Protein	Function
8.0130	DDB_G0282377	*dhkM*	histidine kinase M	Phosphorelay signal transduction
7.9010	DDB_G0291380	*atr1*	Ataxia Telangiectasia and Rad3 related protein kinase	DNA repair
7.6767	DDB_G0282559	*dduA*	Acid phosphatase	Unknown
7.5247	DDB_G0273105	*scdB*	Δ9 fatty acid desaturase ScdB	Lipid metabolism
7.4934	DDB_G0290777		Uncharacterized protein	Unknown
7.2495	DDB_G0286947		Ankyrin repeat-containing protein	Unknown
7.1429	DDB_G0284699	*mtr*	Methionine synthase	Methionine biosynthesis
6.9606	DDB_G0277421		Uncharacterized protein	Unknown
6.9245	DDB_G0294491	*trxB*	Thioredoxin	Oxidation-reduction processes
6.9105	DDB_G0293034		Uncharacterized short-chain dehydrogenase/reductase family member	Unknown
6.8868	DDB_G0293374	*dut*	dUTP diphosphatase, mitochondrial	Nucleotide metabolism
6.8613	DDB_G0269158	*npcA*	Niemann-Pick C type protein	Lipid transport
6.7216	DDB_G0270024	*mgp2*	Mental retardation GTPase activating protein (MEGAP), member 2	Contractile vacuole regulation
6.6999	DDB_G0278473		Uncharacterized transmembrane protein	Unknown
6.6902	DDB_G0285559		Uncharacterized protein	Unknown
6.6048	DDB_G0287461	*abcG3*	ABC transporter G family protein	ATPase activity, coupled to transmembrane movement of substances
6.5830	DDB_G0268966	*lip1*	Lipase family member 1	Lipid metabolism
6.5740	DDB_G0287961		WD40 repeat and DENN domain-containing protein	Regulation of Rab GTPase signaling
6.5114	DDB_G0282579	*pldZ*	Phospholipase D3	Phospholipid metabolism
6.4944	DDB_G0281313		β-galactosidase	Carbohydrate metabolism
6.4761	DDB_G0290753		C2 domain-containing protein	Unknown
6.4595	DDB_G0291996	*gxcU*	Rac guanine nucleotide exchange factor U	Regulation of Rho GTPase signaling
6.4398	DDB_G0272991	*exoc7*	Exocyst complex subunit 7	Contractile vacuole discharge
6.3538	DDB_G0286219		Chromatin remodeling complex subunit R (CHR) protein	Unknown
6.2430	DDB_G0279611		Sterol desaturase family protein	Sterol biosynthesis

Table S9. Hierarchical cluster 5 proteins with a peak of abundance at the culmination and fruiting body stages. The 25 top proteins have been ranked according to their log_2 fold change (LogFC) of abundance at the fruiting body stage relative to all other stages pooled. Information was retrieved from dictyBase. Protein functions, when not determined experimentally, are deduced from homologous proteins in other organisms or from their domain architecture. # Proteins with a signal peptide.

LogFC	Gene ID	Gene name	Protein	Function
9.2639	DDB_G0273601	*sigL*	EGF-like domain-containing protein (p-selectin)	Unknown (extracellular matrix?). Regulated by transcription factor SrfA #
9.2369	DDB_G0286649		Carbohydrate binding domain-containing protein	Cellulose binding #
9.1172	DDB_G0278953		CBS domain-containing protein	Sensor of cellular energy? Expressed in psp cells
9.0658	DDB_G0277141	*cotC*	Spore coat protein SP60	Spore coat integrity. Expressed in psp cells #
8.9413	DDB_G0268028		DUF5923 domain-containing protein	Unknown. Expressed in psp cells
8.6601	DDB_G0270190		Cellulose-binding domain-containing protein	Cellulose binding and hydrolysis. Expressed in pstAO cells #
8.5871	DDB_G0276939	*pspB*	Spore coat protein SP85, Prespore protein B	Spore middle layer formation, in complex with SP65 (cotE) and cellulose. Expressed in psp cells #
8.4675	DDB_G0286647	*ecmD*	Extracellular matrix protein D, sheathin D	Cellulose binding in the extracellular matrix. Expressed in pst cells #
8.2342	DDB_G0291314	*hsp101*	Disaggregase, Heat shock protein 101	Protein aggregation in response to heat stress
8.2119	DDB_G0283575	*gsta5*	Putative glutathione S-transferase α-5	Glutathione-mediated detoxification
8.0203	DDB_G0286055		Peptidase C1A family protein	Unknown #
7.8510	DDB_G0276761	*cotB*	Spore coat protein SP70	Spore outer layer formation. Expressed in psp cells #
7.7614	DDB_G0267898		EGF-like domain-containing protein	Unknown (extracellular matrix?) Expressed on pstAB #
7.7239	DDB_G0274451		Uncharacterized protein	Unknown
7.7041	DDB_G0290557		Thiamine pyrophosphate binding domain-containing protein	Unknown
7.6587	DDB_G0277275	*poxA*	Peroxinectin A	Oxidation-reduction processes. Regulated by transcription factor SrfA #
7.6116	DDB_G0275255		Uncharacterized protein	Unknown. Expressed in pstO cells #
7.5475	DDB_G0290925	*psiD*	PA14 domain-containing protein	Carbohydrate binding. Expressed in psp cells #
7.4916	DDB_G0286025		Cellulose-binding domain-containing protein	Cellulose binding and hydrolysis. Expressed in pstAB, pstA, and pstO cells #
7.4299	DDB_G0268250		Uncharacterized	Unknown #
7.4194	DDB_G0282255		Carbohydrate-binding domain containing protein	Cellulose binding #
7.3835	DDB_G0286285		Uncharacterized protein	Unknown
7.2816	DDB_G0277871	*rsc12*	Uncharacterized protein	Sorocarp development #
7.1132	DDB_G0291291	*ecmF*	Carbohydrate-binding domain-containing protein EcmF	Cellulose binding in the extracellular matrix. Expressed in pst cells. Regulated by transcription factor STATa #
7.0577	DDB_G0292696	*colA*	Colossin A	Unknown

Table S10. Hierarchical cluster 6 proteins with a trough of abundance at the mound stage. The 25 top proteins have been ranked according to their log_2 fold change (LogFC) of abundance at the mound stage relative to all other stages pooled. Information was retrieved from dictyBase. Protein functions, when not determined experimentally, are deduced from homologous proteins in other organisms or from their domain architecture.

LogFC	Gene ID	Gene name	Protein	Function
-8.0636	DDB_G0274587	*nup62*	Nuclear pore protein 62	Structural constituent of the nuclear pore
-7.6543	DDB_G0268630	*spsA*	Spermidine synthase	Polyamine metabolism
-7.3693	DDB_G0282313		Heat shock protein DnaJ family protein	Protein folding
-7.3024	DDB_G0273725	*snf12*	SWI/SNF protein SNF12	Chromatin remodeling
-6.9422	DDB_G0268218		Uncharacterized protein	Unknown
-6.9091	DDB_G0270152	*maspS*	Aspartyl-tRNA synthetase, mitochondrial	Protein biosynthesis
-6.8618	DDB_G0290485	*dlrA*	leucine-rich repeat-containing protein DlrA	Unknown
-6.8425	DDB_G0270116		Uncharacterized protein	Unknown
-6.7635	DDB_G0274657		Glutaredoxin-related family protein	Cell redox homeostasis
-6.6779	DDB_G0276103	*enlA*	Enlazin, Fimbrin 2	Actin cytoskeleton remodeling
-6.5921	DDB_G0283007		CUE domain-containing protein	Unknown
-6.5050	DDB_G0278453		Coiled-coil domain-containing protein	Unknown
-6.4896	DDB_G0280235		DUF1682 family protein	Unknown
-6.4890	DDB_G0270806	*ldhd*	D-lactate dehydrogenase	Metabolism
-6.2732	DDB_G0290913	*mcfS*	Mitochondrial substrate carrier family protein S translocase	Transport of carnitine across the mitochondrial membrane
-6.2496	DDB_G0349279		Uncharacterized transmembrane protein	Unknown
-6.2353	DDB_G0270358		UBX domain-containing protein 7	Unknown
-6.2314	DDB_G0288203		Armadillo repeat-containing protein	Unknown
-5.9802	DDB_G0272092		Tyrosine kinase-like and ankyrin repeat-containing protein	Intracellular signal transduction
-5.9765	DDB_G0273855		Short-chain dehydrogenase/reductase family protein	Unknown
-5.9685	DDB_G0282539	*nagB*	N-acetylglucosaminidase	Carbohydrate metabolism
-5.9052	DDB_G0288479	*lsm8*	U6 snRNA-associated Sm-like protein	mRNA splicing
-5.8937	DDB_G0277841	*ctr9*	RNA polymerase II complex component	Regulation of transcription and histone methylation
-5.6977	DDB_G0292034	*mcfP*	Mitochondrial substrate carrier family protein P	Transport of molecules across the mitochondrial membrane
-5.6488	DDB_G0270470		Tetratricopeptide TPR-1 repeat-containing protein	Unknown

Table S11. Hierarchical cluster 7 proteins with a peak of abundance at the fruiting body stage only. The 25 top proteins have been ranked according to their log_2 fold change (LogFC) of abundance at the fruiting body stage relative to all other stages pooled. Information was retrieved from dictyBase. Protein functions, when not determined experimentally, are deduced from homologous proteins in other organisms or from their domain architecture. # Proteins with a signal peptide.

LogFC	Gene ID	Gene name	Protein	Function
12.8159	DDB_G0274337		Uncharacterized protein	Unknown
12.3558	DDB_G0288323	*strM*	BAR domain-containing protein	Lipid binding and curvature sensing
11.7875	DDB_G0284677	*expl2*	Expansin-like protein 2 (Ddexpl2)	Modification of cell wall to allow expansion during growth #
10.9082	DDB_G0274317		BAR domain-containing protein	Uncharacterized. Lipid binding and curvature sensing?
10.5714	DDB_G0288489	*spoA*	Spore-specific protein A	Unknown
10.5182	DDB_G0285289	*spoB*	Spore-specific protein B	Unknown
9.8391	DDB_G0279023		Unknown	Unknown #
9.7897	DDB_G0271134	*celA*	Cellulase 270-6	Spore germination. Expressed in psp cells #
9.6942	DDB_G0268248		Uncharacterized protein	Unknown #
9.6265	DDB_G0285273		Alkyl hydroperoxide reductase/thiol specific antioxidant family protein	Cell redox homeostasis
9.5864	DDB_G0274433		CBS domain-containing protein	Sensor of cellular energy?
9.4997	DDB_G0287391	*ranbp1*	Ran-binding protein 1	Protein import into the nucleus
9.4159	DDB_G0294473	*staA*	Stalk-specific protein A	Cellulose binding. DIF-inducible, stalk specific #
9.4030	DDB_G0287549		Spore germination protein 1/2/3-related protein	Spore germination #
9.3797	DDB_G0295797		EGF-like domain-containing protein	Unknown #
9.2371	DDB_G0290245	*psiC*	PA14 domain-containing protein	Carbohydrate binding. Expressed in psp cells #
9.1482	DDB_G0288201		CBS domain-containing protein	Sensor of cellular energy?
9.0286	DDB_G0267476	*sigK*	EGF-like domain-containing protein	Uncharacterized. Regulated by transcription factor SrfA. Expressed in stalk cells. Extracellular matrix? #
8.8390	DDB_G0286563	*sigM*	EGF-like domain-containing protein	Unknown. Regulated by transcription factor SrfA. Extracellular matrix? #
8.5095	DDB_G0270288		Carbohydrate-binding domain-containing protein	Cellulose binding? #
8.4497	DDB_G0271314	*iliH*	Endoglucanase	Cellulose binding and hydrolysis #
8.3567	DDB_G0277903	*cotE*	Spore coat protein SP65	Spore outer layer formation, in complex with SP85 (pspB). Expressed in cup cells and spores. Regulated by transcription factor BzpF #
8.2692	DDB_G0293442		Uncharacterized protein	Unknown
8.1250	DDB_G0284261	*nxnB*	Annexin C2	Phospholipid/calcium binding
7.9331	DDB_G0269112	*celB*	Cellulose-binding protein	Cellulose binding. Expressed in psp cells #

Table S12. Hierarchical cluster 8 proteins with a peak of abundance at the culmination stage. The 25 top proteins have been ranked according to their $\log_2$ fold change (LogFC) of abundance at the culmination stage relative to all other stages pooled. Information was retrieved from dictyBase. Protein functions, when not determined experimentally, are deduced from homologous proteins in other organisms or from their domain architecture. # Proteins with a signal peptide.

LogFC	Gene ID	Gene name	Protein	Function
8.1886	DDB_G0291253	*dia2*	Differentiation-associated protein 2 (DIA-2)	Unknown #
7.4751	DDB_G0269280		Uncharacterized protein	Unknown
7.4504	DDB_G0287009	*mcfC*	Mitochondrial substrate carrier family protein C	Calcium-dependent ATP-Mg/Pi carrier
7.1937	DDB_G0283611	*cbpF*	Calcium-binding protein F	Unknown
7.0752	DDB_G0272827	*cbpI*	Calcium-binding protein I	Unknown. Expressed in psp cells.
6.8159	DDB_G0292160		Ortholog of human LAMTOR	Regulation of lysosomal signaling and trafficking
6.6238	DDB_G0289833	*snxA*	Sorting nexin A	Endocytosis
6.4969	DDB_G0291406		Ankyrin repeat and helicase domains-containing protein	Unknown
6.4780	DDB_G0285971	*rpl37*	60S ribosomal protein L37	Structural component of the ribosome
6.2967	DDB_G0270218	*glkA*	Probable serine/threonine-protein kinase GlkA	Regulation of growth, chemotaxis and multicellular development
6.2364	DDB_G0276415	*rps18*	40S ribosomal protein S18	Structural component of the ribosome
6.1089	DDB_G0276759	*cbpA*	Calcium-binding protein A , CBP1	Actin cytoskeleton remodeling
6.0774	DDB_G0283613	*cbpC*	Calcium-binding protein C	Actin cytoskeleton remodeling?
5.9293	DDB_G0281993		Uncharacterized protein	Unknown
5.9102	DDB_G0274299		Uncharacterized protein	Unknown. Expressed in pstAB cells
5.8815	DDB_G0290595		CBS domain-containing protein	Sensor of cellular energy?
5.8801	DDB_G0293802		Uncharacterized transmembrane protein	Unknown #
5.6542	DDB_G0283961		Uncharacterized transmembrane protein	Unknown #
5.5812	DDB_G0290035		Aminotransferase class V domain-containing protein	Unknown
5.5414	DDB_G0283609	*cbpG*	Calcium-binding protein G	Unknown
5.4734	DDB_G0286171	*uqcrb*	Cytochrome bd ubiquinol oxidase, 14 kDa subunit	Mitochondrial respiratory chain
5.4613	DDB_G0268854	*gnt4*	GlcNAc transferase	Protein glycosylation
5.4608	DDB_G0289883	*cupG*	Calcium up-regulated protein G	Unknown
5.4112	DDB_G0278017		Profilin superfamily protein	Actin cytoskeleton remodeling
5.3465	DDB_G0282245	*forD*	Formin D	Actin cytoskeleton remodeling

Supplementary figures

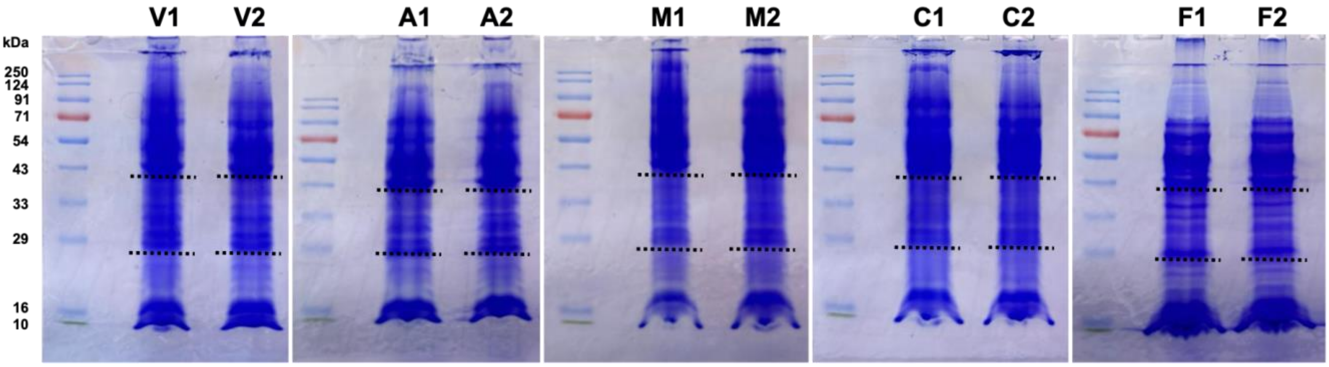

Figure S1. *Dictyostelium discoideum* proteins across the life cycle. Samples (100 µg) were resolved on 12% SDS–PAGE gels and stained with Coomassie blue. Vegetative, aggregation, mound, culmination and fruiting body stages are indicated as V, A, M, C and F, respectively. Biological replicates are denoted as 1 and 2. Black dotted lines indicate the gel lane fractions (high, medium and low molecular weight) excised for in-gel digestion.

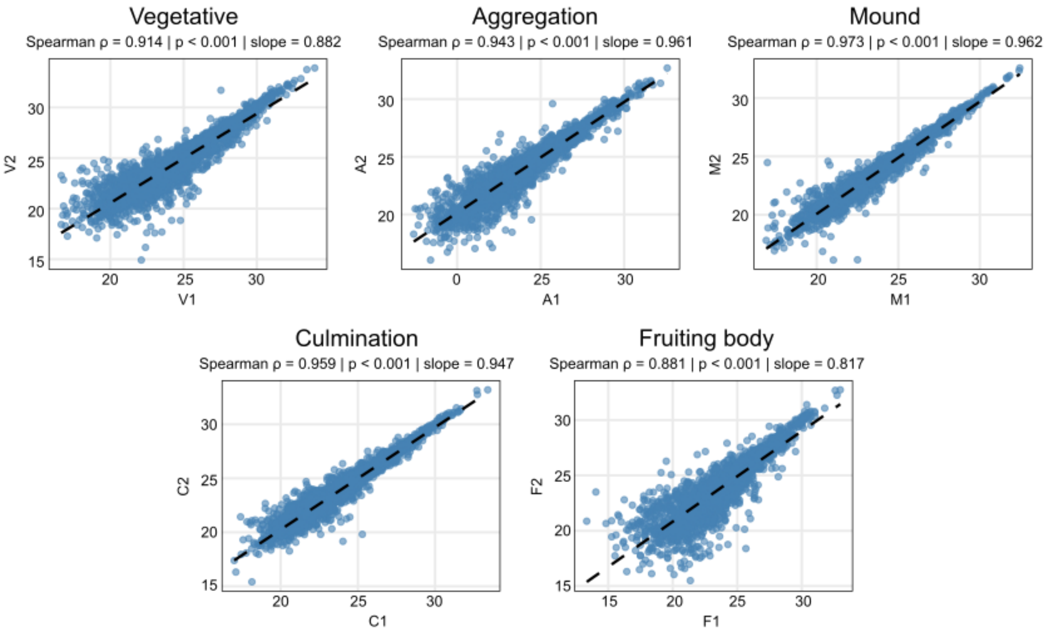

Figure S2. Spearman correlation between biological duplicates for each developmental stage. Raw, non-imputed data for which both replicates were available were used for the analysis. The x- and y-axes represent log_2-transformed protein abundance values. P < 0.001 for all stages. Number of proteins analyzed: 3075 (vegetative), 2868 (aggregation), 2620 (mound), 2940 (culmination) and 2318 (fruiting body).

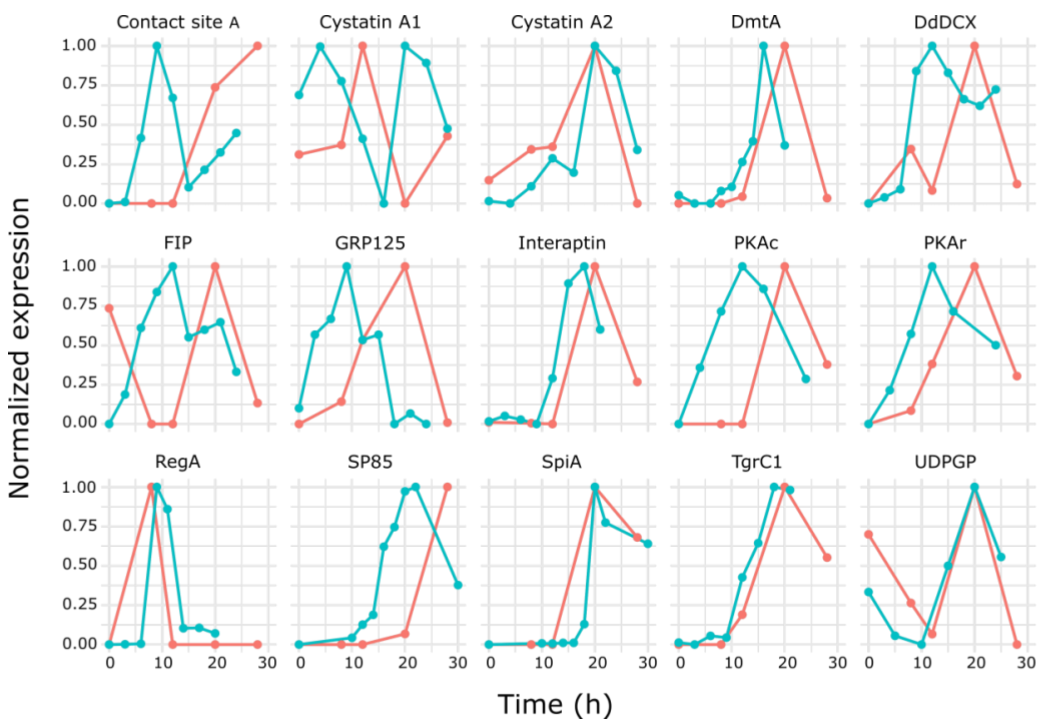

Figure S3. Validation of LC-MS/MS protein abundances using published data. Band intensities on Western blots were quantified using ImageJ. Enzymatic activities for DmtA, PKAc, PKAr and UDPGP were read from published graphs. ● LC-MS/MS protein abundances, ● published data. UDPGP enzyme activity was compared to the sum of the abundances of the two proteins responsible for the activity, encoded by *uppA* and *ugpB*. Both datasets were normalized to a min–max scale of 0 to 1 to make trends across the developmental time series comparable. The x-axis indicates developmental time points and the y-axis represents min–max normalized expression. Sources of the published data are presented in Table S2.

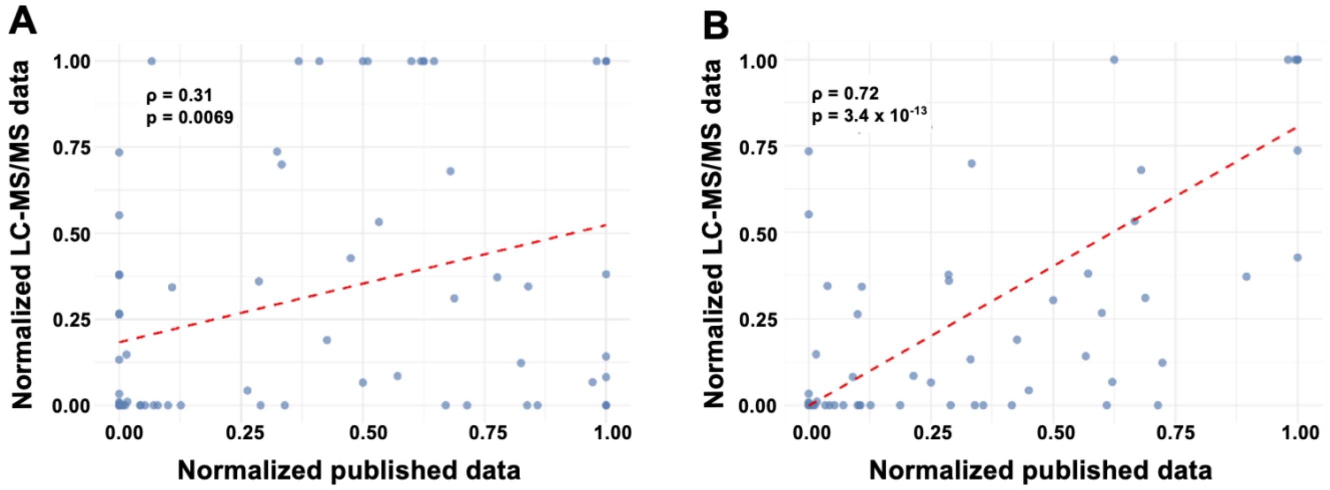

Figure S4. Spearman correlation between LC-MS/MS and published data. A. The min–max normalized data of Figure S2 was used at time points 0, 8, 12, 20 and 28 hours. Values of published data were interpolated from graphs where those exact time points were not available. B. Published data was time-adjusted to align with the LC-MS/MS measurements to match the peak values. Each set of 75 data points was used to calculate the Spearman correlation coefficient ρ and the P value.

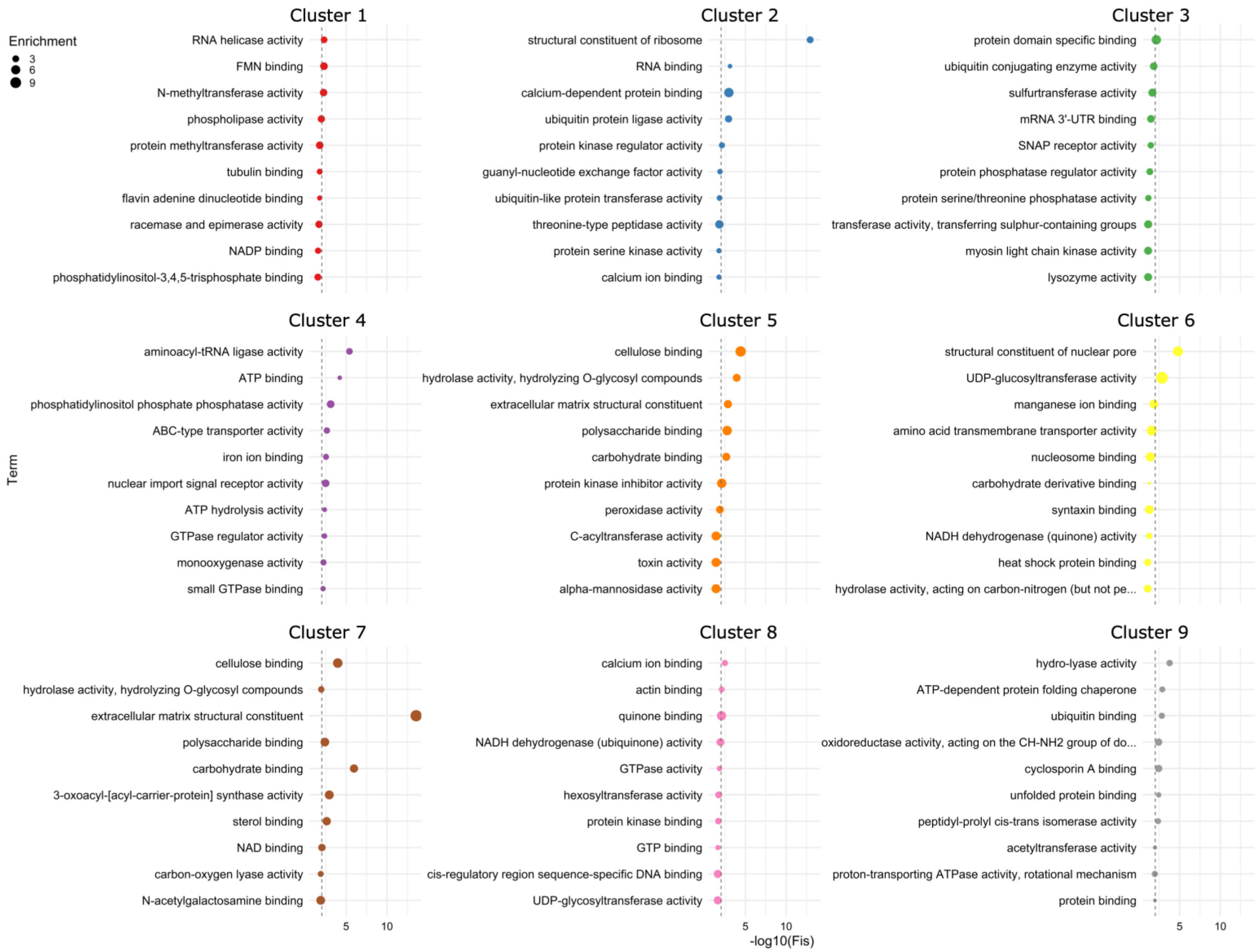

Figure S5. Gene Ontology (GO) molecular function enrichment analysis for each hierarchical cluster of differentially abundant proteins during development (see Figure 5). The ten most significantly enriched GO terms are shown per cluster. P values were calculated using Fisher' s exact test; the vertical dashed line indicates significance at $p < 0.01$ ($-\log_{10}$ scale). Bubble size reflects the enrichment score of each GO term.

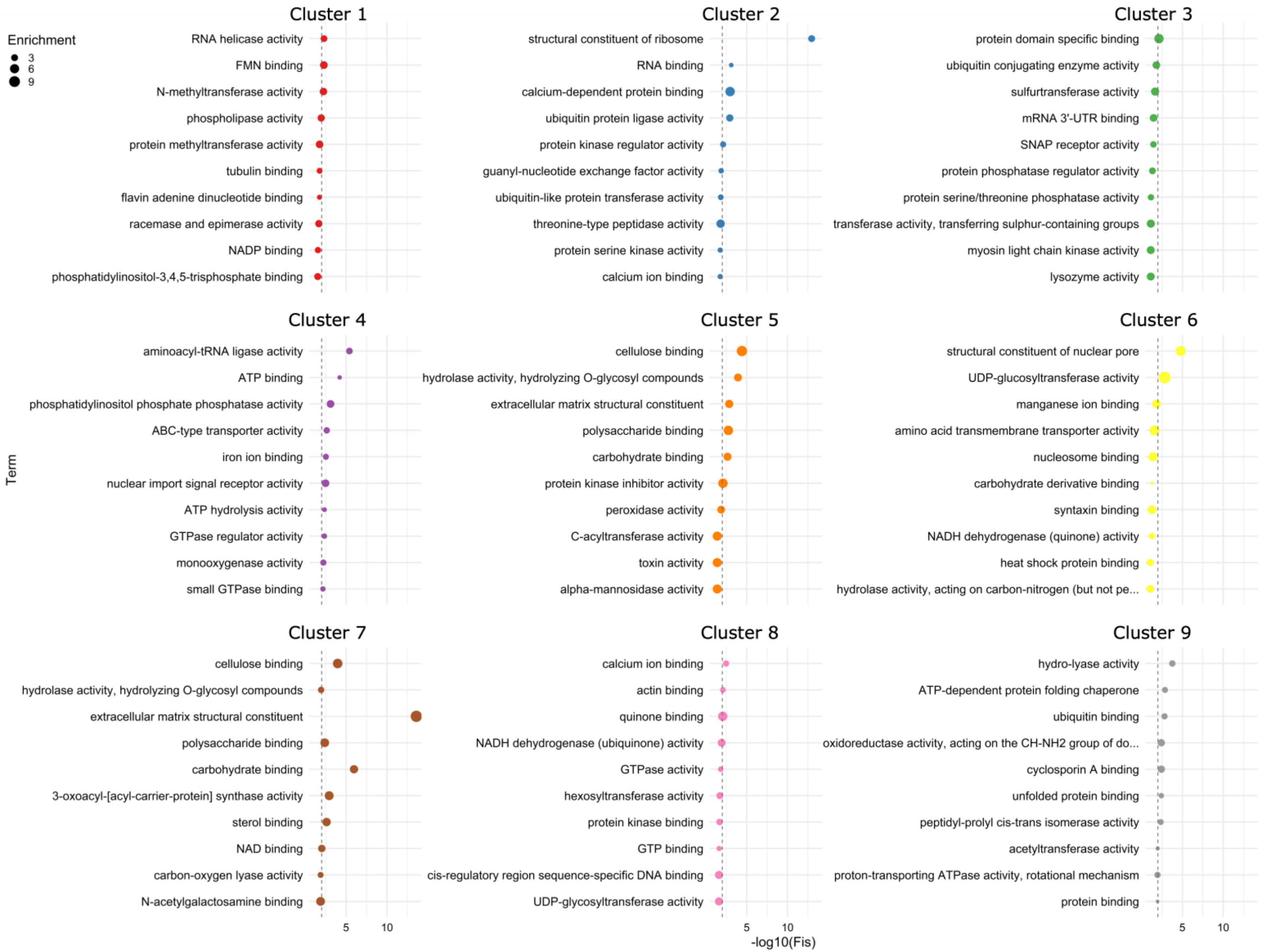

Figure S6. Gene Ontology (GO) cellular component enrichment analysis for each hierarchical cluster of differentially abundant proteins during development (see Figure 5). The ten most significantly enriched GO terms are shown per cluster. P values were calculated using Fisher’ s exact test; the vertical dashed line indicates significance at $p < 0.01$ ($-\log_{10}$ scale). Bubble size reflects the enrichment score of each GO term.

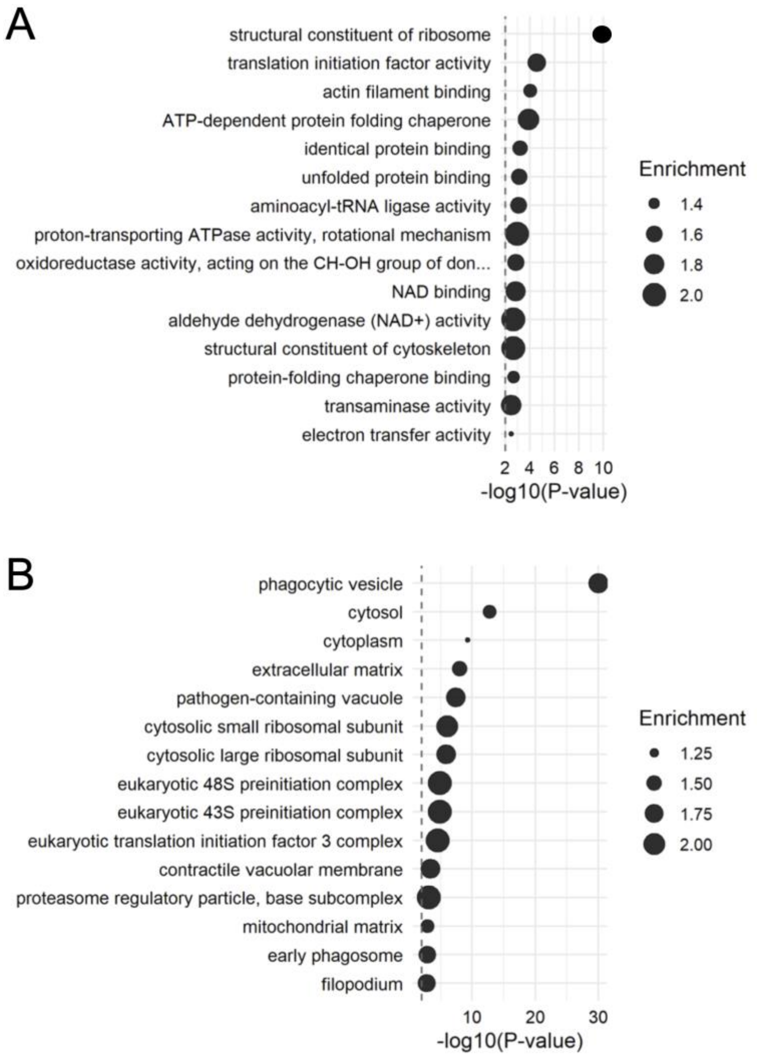

Figure S7. The *D. discoideum* continuous expression proteome. Gene Ontology (GO) molecular function (A) and cellular component (B) enrichment analysis. Enrichment analysis was performed using the clusterProfiler package in R. The top 15 most significantly enriched GO terms are shown. Bubble size represents the enrichment score for each GO term, and statistical significance was assessed using Fisher's exact test; a dashed vertical line marks $p < 0.01$ ($-\log_{10}$ scale).

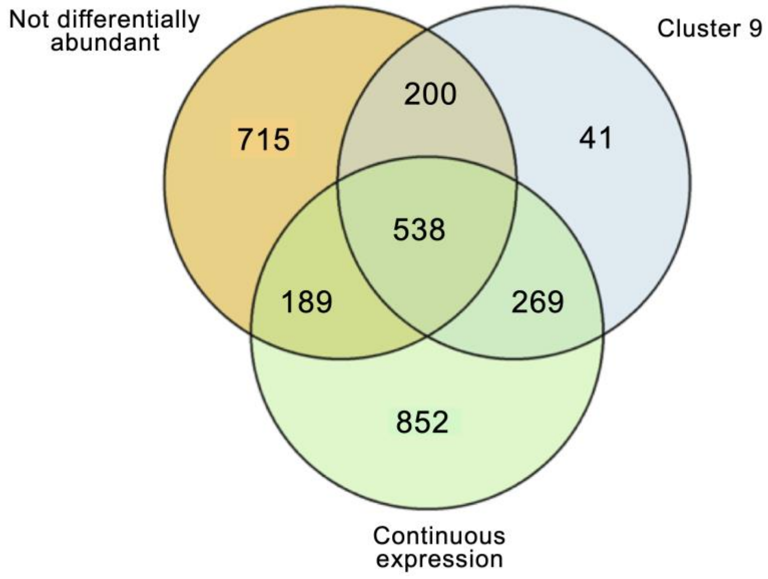

Figure S8. Comparison of hierarchical cluster 9 with the continuous expression proteome and the not differentially abundant proteins ($\log_2$FC not significantly changed).
